# Reduction of oxytocin-containing neurons and enhanced glymphatic activity in the hypothalamic paraventricular nucleus of patients with type 2 diabetes mellitus

**DOI:** 10.1186/s40478-023-01606-w

**Published:** 2023-07-03

**Authors:** Felipe Correa-da-Silva, Martin J. Kalsbeek, Femke S. Gadella, Jorn Oppersma, Wei Jiang, Samantha E. C. Wolff, Nikita L. Korpel, Dick F. Swaab, Eric Fliers, Andries Kalsbeek, Chun-Xia Yi

**Affiliations:** 1 Department of Endocrinology and Metabolism, Amsterdam Gastroenterology, Endocrinology and Metabolism, Amsterdam University Medical Centers, Location AMC, University of Amsterdam, Meibergdreef 9, 1105 AZ Amsterdam, The Netherlands; 2 Laboratory of Endocrinology, Amsterdam Gastroenterology, Endocrinology and Metabolism, Amsterdam University Medical Centers, Location AMC, University of Amsterdam, Amsterdam, The Netherlands; 3 Netherlands Institute for Neuroscience, Amsterdam, The Netherlands

**Keywords:** Hypothalamus, Oxytocin, Type 2 diabetes mellitus, Astrocytes, Glymphatic, Insulin resistance, Microglia

## Abstract

Evidence from animal experiments has shown that the hypothalamic paraventricular nucleus (PVN) plays a key role in regulating body weight and blood glucose levels. However, it is unclear whether neuron populations in the human PVN are involved in the development of type 2 diabetes mellitus (T2DM). To address this, we investigated the neuronal and glial populations in the PVN of 26 T2DM patients and 20 matched controls. Our findings revealed a significant reduction in oxytocin (Oxt) neuron density in the PVN of T2DM patients compared to controls, while other neuronal populations remained unchanged. This suggests that Oxt neurons may play a specific role in the pathophysiology of T2DM. Interestingly, the reduction in Oxt neurons was accompanied by a decreased melanocortinergic input in to the PVN as reflected by a reduction in alpha-MSH immunoreactivity. We also analysed two glial cell populations, as they are important for maintaining a healthy neural microenvironment. We found that microglial density, phagocytic capacity, and their proximity to neurons were not altered in T2DM patients, indicating that the loss of Oxt neurons is independent of changes in microglial immunity. However, we did observe a reduction in the number of astrocytes, which are crucial for providing trophic support to local neurons. Moreover, a specific subpopulation of astrocytes characterized by aquaporin 4 expression was overrepresented in T2DM patients. Since this subset of astrocytes is linked to the glymphatic system, their overrepresentation might point to alterations in the hypothalamic waste clearance system in T2DM. Our study shows selective loss of Oxt neurons in the PVN of T2DM individuals in association with astrocytic reduction and gliovascular remodelling. Therefore, hypothalamic Oxt neurons may represent a potential target for T2DM treatment modalities.

**Supplementary Information:**

The online version contains supplementary material available at 10.1186/s40478-023-01606-w.

## Introduction

Disruptions in the hypothalamic neurocircuitry that controls energy metabolism are associated with the development and progression of metabolic disorders, such as type 2 diabetes (T2DM) and obesity [53]. Initial studies on postmortem human brain tissues pointed to a neuropeptidergic imbalance in the hypothalamus of T2DM individuals, affecting neurons that control appetite curbing [30] and circadian rhythms [24]. Several lines of evidence in experimental rodents indicate a protective role of the paraventricular nucleus of the hypothalamus (PVN) in neuroendocrine, autonomic and behavioural responses to metabolic challenges [34, 35, 38, 57]. However, despite abundant evidence of the participation of the PVN in glycaemic control [27, 60], it remains unclear whether PVN neurons are affected in T2DM individuals.

Proper neuron-glia interactions are necessary for the optimal coordination of feeding behaviour and energy homeostasis [13, 20, 43]. Animal studies have shown that a prolonged obesogenic diet triggers hypothalamic reactive gliosis, which is linked to numeric and functional loss of neurons in control of metabolism [53]. Specifically, the lack of astrocytic trophic support associated with microglia-driven local inflammation is considered a key mechanistic node in the progression of metabolic diseases, such as obesity and diabetes [46]. Although the importance of disruptive neuron-glia interactions is broadly reported in experimental rodents, it remains unknown to what extent these findings can be translated to the human condition. Furthermore, angiogenesis, referring to the formation of new blood vessels from pre-existing ones, plays a crucial role in the progression of diabetic retinopathy [3, 14]. Our previous study found an increased number of arterioles in the infundibular nucleus (IFN) of individuals with T2DM, suggesting that angiogenesis can occur in the human hypothalamus during T2DM [56]. Whether T2DM-associated angiogenesis also takes place in other hypothalamic regions, including the PVN, remains a question.

In this study, we investigated the neuron-glia-vasculature in the PVN of control and T2DM individuals, including neurons expressing oxytocin (Oxt), arginine-vasopressin (AVP), or corticotrophin releasing hormone (CRH), microglia, two subpopulations of astrocytes, and lastly, arteries and arterioles. We found a selective and marked reduction in Oxt-immunoreactive (Oxt-ir) neurons, in association with gliovascular remodelling that may facilitate and contribute to the progression of T2DM.

## Methods

### Subject information

Postmortem hypothalamic tissue specimens of 20 non-diabetic controls and 26 T2DM subjects were obtained from the Netherlands Brain Bank (NBB), through autopsy approved by the Medical Ethic Committee of the VU Medical Center, the Netherlands. These T2DM and control subjects were matched for age, sex, postmortem delay, and tissue fixation time. Furthermore, patients with known neurological or psychiatric disorders were excluded. The donors or their next of kin gave consent for a brain autopsy, access to medical records and utilization of the brain tissue for research purposes. Individuals considered controls were defined as not having any known endocrine or metabolic pathologies. Patients who experienced corticosteroids medication prior to death were excluded from the study. An overview of clinico-pathological details of the subjects can be found in Table 1.

**Table 1:** Anthropometric data and clinical information

NBB	Sex	Age (years)	PMD (hours)	Fixation time (days)	BMI	pH csf	Post-abs. Glucose	HbA1c	Insulin treatment	Cause of death and clinical diagnosis
*Non-diabetic controls*										
1991–205	F	65	9,50	/	20	/	/	/	/	Mamma tumor, cardiac failure
1997–065	F	76	14,50	27	32	/	/	/	/	Myocardial infarction, unilateral nephrectomy and adrenalectomy
1997–100	M	76	19,00	/	25	/	/	/	/	Septic syndrome after complicated implantation aorta bifurcation prosthesis due to juxta renal aneurysm
1997–127	F	49	13,50	/	25	/	/	/	/	Respiratory insufficiency with ascites and sputum retention due to metastasised cervix carcinoma
1997–146	F	100	24,00	62	30	/	4,4	/	/	Pneumonia, myocardial infarction, dyspnea
1998–035	F	65	20,00	55	27	/	/	/	/	Mesenterial ischemia complications, dyspnea, atrial fibrillation
2000–072	M	78	18,00	45	40	5,84	1,4	/	/	Kidney failure, dehydration, heart failure, renal insufficiency
2001–021	M	82	7,67	32	28	6,07	/	/	/	Heart attack, ischemic heart disease, kyphosis of backbone
2001–069	F	68	5,75	32	25	6,97	/	/	/	Legal euthanasia, vaginal carcinoma, kidney tumor and lung metastasis
2007–088	F	82	5,17	61	22	6,8	4,3	/	/	Cachexia, cardiac failure, encephalopathy, mitral valve insufficiency
2008–052	F	62	7,92	/	21	6,4	/	/	/	Euthanasia
2009–022	F	77	2,92	39	33	7,06	/	/	/	Pulmonary metastasis of vulva carcinoma
2009–039	F	82	12,92	38	28	6,21	7,4	/	/	Heart failure, prostate carcinoma
2009–095	F	71	7,17	53	33	6,31	/	31	/	Renal failure, CVA, hypertensive retinopathy
2010–008	M	81	4,50	/	20	6,3	/	31	/	Terminal pancreas carcinoma
2010–013	M	70	6,25	68	26	6,45	/	41	/	Acute myocardial infarction, prostate carcinoma
2011–082	F	84	5,92	44	39	6,1	6,2	42	/	Respiratory failure, angina pectoris, mitral valve insufficiency
2012–005	F	84	5,60	57	31	6,68	7,5	/	/	Heart failure, metastatic breast cancer, scoliosis
2012–033	F	95	5,67	69	28	6,46	/	/	/	Heart failure, cachexia and dehydration, pulmonary disease
2012–104	M	79	6,50	67	31	6,71	7,4	/	/	Legal euthanasia, ischemic colitis, heart failure with dyspnoea
*T2DM subjects*										
1998–080	F	72	24,00	67	/	/	12	/	/	Cardiac decompensation, complete respiratory insufficiency
1998–112	F	84	9,33	28	/	/	/	/	/	Pulmonary emboli, CVA, atherosclerosis
1998–126	M	71	6,00	41	26	6,54	6,3	/	/	Respiratory insufficiency, lung carcinoma
2001–003	M	69	10,00	31	/	6,37	10,7	/	/	Cardiac arrest, urinary tract infection and fever, cholelithiasis
2001–061	F	85	4,33	33	/	6,35	5,4	/	/	Myocardial infarction, parkinsonism and depression
2003–054	M	67	4,50	50	/	/	/	126	/	Cardiac shock, CVA
2005–027	F	64	4,33	38	/	6,45	8,8	40	/	Respiratory failure, CVA
2007–061	F	83	5,33	48	/	6,07	5,1	/	/	Cachexia, CVA, arteriosclerosis
2010–046	F	88	6,50	/	22	5,93	/	/	/	Dysregulated Diabetes mellitus type II and Dehydration
2011–027	M	80	3,30	44	24	6,16	5,5	44	/	Pneumonia, CVA, ischemic attack
2012–049	F	70	7,58	59	/	6,03	/	/	/	Cachexia, pancreas carcinoma
2012–088	F	85	6,42	49	29	6,6	9,2	/	/	Legal euthanasia, hypoparathyroidism
1989–032	M	84	5,08	29	/	6,77	/	/	Yes	Heart failure, intestinal tumour
1995–078	F	80	6,25	34	/	6,96	/	/	Yes	Dehydration, angina pectoris
1998–056	F	83	5,25	/	/	7,3	/	/	Yes	Coloncarcinoma, Euthanasia
1999–015	F	93	2,58	36	/	6,6	/	/	Yes	Pneumonia, dehydration, breast cancer
2004–085	F	71	4,58	44	/	/	5,8	/	Yes	Dehydration, CVA with right side paresis
2006–033	M	79	5,00	48	/	6,37	6,6	/	Yes	Pneumonia, dehydration, CVA, choledochus carcinoma
2008–061	F	62	5,00	65	/	6,11	8	79	Yes	Cachexia, hyperthyroidism
2008–105	F	89	3,87	58	30	7,30	6,6	44	Yes	Pneumonia, coronary artery bypass, atrial fibrillation
2009–096	M	92	8,42	53	/	6,14	6,7	/	Yes	Heart failure, myocardial infarction
2010–092	M	85	5,08	44	16	6,17	/	/	Yes	Dehydration and cachexia, CVA
2012–092	M	90	5,75	49	/	6,35	/	/	Yes	Prostate carcinoma
2012–118	M	96	4,17	68	23	6,09	/	45	Yes	Transient ischemic attack, urinary tract infection, diabetic retinopathy
2014–051	M	92	7,75	47	29	6,55	/	49	Yes	Liver cirrhosis ascites and anuria, hepatic cirrhosis
2014–063	F	93	7,58	/	22	6,27	/	/	Yes	Heart failure

NBB: Netherlands Brain Bank; PMD: postmortem delay; BMI: Body mass index; Post abs.: post absorptive; CVA: cardiovascular accident

### Anatomical identification

After autopsy, the hypothalami were immersed in 10% phosphate-buffer formalin at room temperature. After fixation, brain tissue was embedded in paraffin and sectioned in a rostral to caudal orientation at 6 μm thickness. The anatomical orientation of PVN was determined by Nissl staining of every 100th section available, and further analysed by evaluating the range of Oxt-ir. For histological procedures, sections were mounted on Superfrost^+^ slides, dried on a 37 °C heating plate for 48 h. To remove the paraffin, the slides were immersed in 100% xylene, rehydrated in grading ethanol (100%–50%), and rinsed in distilled water.

### Immunohistochemistry and immunofluorescence

As the peak level of the Oxt-ir, AVP-ir and CRH-ir neurons along the rostral-caudal axis of the PVN is almost identical (data not shown), all the immunohistochemistry analysis was performed in sections adjacent to the one with the highest count of Oxt-immunoreactive neurons. To unmask epitopes, heat induced antigen retrieval was performed using microwave treatment (10 min at 700 W). The requirement and conditions of antigen retrieval were determined in pilot studies (data not shown). Incubation and antigen retrieval steps for ionized calcium-binding adapter molecule 1 (Iba1), CRH, cluster of differentiation 68 (CD68)/Iba1, alpha-smooth muscle actin (alpha-SMA) were performed in citrate buffer (82.5 mM sodium citrate dihydrate and 17.5 mM citric acid; pH 6.0). Incubation and antigen retrieval for alpha-melanocortin stimulating hormone (alpha-MSH), co-labelling of Oxt/Iba1 or AVP/Iba1 were performed in Tris buffer (1 M, pH 9.0). Antigen retrieval was not required for AVP, Aq4, GFAP, and Oxt single immunohistochemical staining.

After cooling, sections were washed in Tris-buffer saline (TBS, 50 mM Tris–Cl and 150 mM NaCl, pH 7.6) and treated with 3% hydrogen peroxide in SUMI buffer (0.25% gelatine, 0.5% Triton X-100 in TBS (pH 7.6)). The sections were then washed in TBS and incubated with primary antibody for one hour at room temperature followed by overnight incubation at 4 °C. The next day, sections were rinsed and incubated with biotinylated secondary antibody and avidin–biotin complex (1:400 horse anti-rabbit IgG, goat anti-rat IgG, or rabbit anti-sheep IgG, Vector Laboratories; according to the species in which the primary antibody was raised). The product was visualized by incubation in 0.5 mg/mL 3,3’-Diaminobenzidine (Sigma Chemical Co.) and 0.01% hydrogen peroxide (Merck).

To perform immunofluorescence, sections for staining CD68/Iba1, AVP/Iba1, Oxt/Iba1 or alpha-SMA were incubated with biotinylated goat anti-mouse IgG (against CD68, AVP or Oxt) (1:400, Vector Laboratories) for one hour. The sections were then rinsed and incubated with a corresponding fluorescent secondary anti-rabbit antibody (against iba1) and streptavidin-fluorescence for one hour. Sections were then rinsed with TBS, followed by a DAPI counterstaining (1:5000, 62,248, ThermoFischer). A list of primary antibodies used in our experiments can be found in Additional file 1: Table S1.

### Images acquisition and quantitative analysis

The immunohistochemically stained sections were captured using an Axio Scanner (Zeiss) and analysed with FIJI and/or QuPath software. Analysis of Oxt-ir, AVP-ir and CRH-ir neurons was performed by outlining the total area covered by positive signal in two consecutive sections. Total area outlined, number of particles, particle size and area of coverage of positive signal were measured through the employment of the software tool “particle analysis” (FIJI). Neuronal soma was considered particles with areas between 30 and 300 µm^2^; and the total soma number obtained was divided by total area, resulting in soma number/mm^2^. The average soma size was obtained by calculating the mean area of the positive particles. Relative area of coverage of positive particles was calculated by determining the percentage of area of positive signal in relation to the total area.

**Fig. 1:**
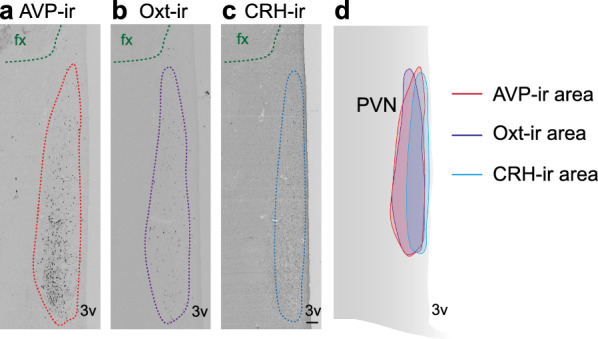
An overview of AVP-ir, Oxt-ir and CRH-ir neurons in the PVN of the same subject. **a** Representative image of AVP-ir neurons distribution in the PVN; **b** representative image of Oxt-ir neurons distribution in the PVN and **c** representative image of CRH-ir neurons distribution in the PVN. fx: fornix; 3v: third ventricle. **d** A diagram depicts the human hypothalamus showing the areas of AVP-ir, Oxt-ir and CRH-ir. Scale bar: 250 μm

To assess glial markers and alpha-MSH-ir, consecutive sections were examined, focusing on those that had the highest number of Oxt-ir neurons. Given the significant overlap between Oxt-ir/AVP-ir areas and CRH-ir covered area (as shown in Fig. 1), in contrast to their largely separate compartments within the rodents’ PVN [10] and the lack of observed changes in CRH-ir neurons, the corresponding areas covered by Oxt-ir neurons in adjacent sections were outlined for quantification of these markers.

For microglia and astrocytes, a similar strategy was employed. Microglial (Iba1-ir) and astrocytic (GFAP-ir and Aq4-ir) soma were considered particles with areas ranging from 20 µm^2^—100 µm^2^ according to previously published material [30] and pilot studies (data not shown). Alpha-MSH-ir relative area of coverage was obtained through a masked representation of the staining using the “pixel classification” tool in QuPath. The relative microglial area surrounding AVP-ir and Oxt-ir neurons was calculated using QuPath software [7].

In brief, the positive signal for neurons and microglia was masked using the “pixel classification” tool. Masked signal ranging from 30 and 300 µm^2^ and minimum hole size of 100 µm^2^ were used as criteria to create objects reflecting neuronal staining. Following this, a circular area of 10 µm^2^ radius was created for every neuronal annotation using the “expand annotation” tool. Then, a masked signal for Iba1-ir was created, but no minimum particle area or hole size was imposed. The area of Iba1-ir signal was calculated within every expanded object and averaged by subject.

Images from immunofluorescent experiments were acquired using a SP8 SMD confocal microscope (Leica) for Iba1/CD68 co-labelling or Axio Scanner (Zeiss) for Oxt and AVP/Iba1 co-labelling, and single alpha-SMA. Analysis of 3D volumes of Iba1-ir and CD68-ir was performed in Imaris 9.0. Specifically, a surfaced rendering of each marker was generated for individual cells and the CD68/Iba1 ratio was calculated. An averaged value of this ratio was used for each subject. For images acquired using the Axio Scanner, pilot sections were used to determined laser intensity for each marker. All sections of the same marker were acquired consecutively within a manually outlined region of interest.

### Statistical analysis

Data are expressed as mean ± SEM. All markers evaluated passed a normality test (D’Agostino and Pearson test) and statistical comparisons were performed by Student`s t test and a p value bellow 0.05 was considered significant. Mean values, standard deviation and associated *p* values can be found in Additional file 1: Tables S2 and S3. All statistic tests were performing using GraphPad Prism 8.12.

## Results

### Selective reduction of oxytocin neurons in the PVN of the T2DM individuals

**Fig. 2:**
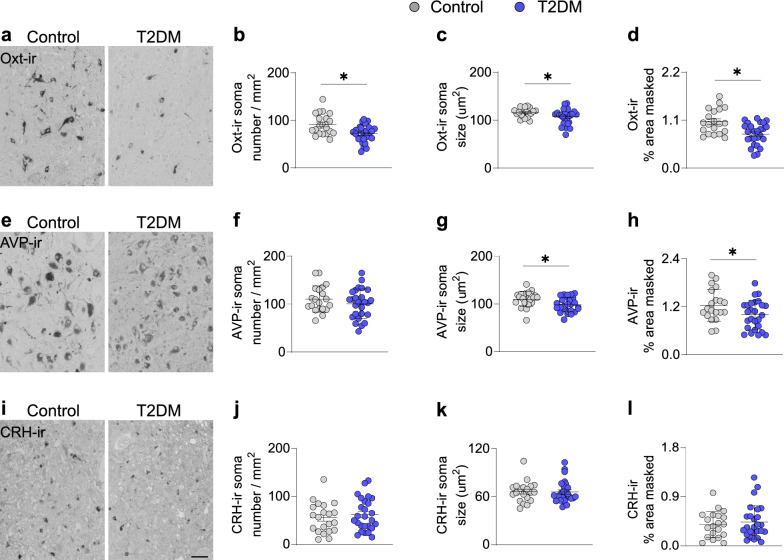
Selective reduction of Oxt-ir in the PVN of T2DM subjects. **a** Representative images of Oxt-ir neurons in the PVN of control (n = 20) and T2DM (n = 26) subjects. Plot of neural parameters specifying **b** Oxt-ir soma number / mm^2^ (neuronal density), **c** Oxt-ir average soma size and **d** relative masked area by the Oxt-ir cells. **e** Representative images of AVP-ir neurons in the PVN of control and T2DM subjects. Quantitative analysis of **f** AVP-ir soma number / mm^2^ (neuronal density), **g** AVP-ir average soma size and **h** relative masked area by the AVP-ir cells. **i** Representative images of CRH-ir neurons in the PVN of control and diabetic subjects. Plot of **j** CRH-ir soma number / mm^2^ (neuronal density), **k** CRH-ir average soma size and **l** relative masked area by the CRH-ir cells. Note that Oxt-ir neurons are drastically reduced in the PVN of diabetic subjects, whereas AVP- and CRH-containing neurons are unaltered in number, suggesting a selective effect of T2DM pathophysiology in Oxt neurons. Also note that the soma size of the CRH neurons is substantially smaller than the Oxt and AVP neurons, as CRH neurons only belong to the parvocellular component of PVN. Scale bar: 50 µm in **a**, **e** and **i**. Data are represented as mean ± SEM. *p < 0.05. Significance was calculated using Student`s t test in **b**–**d**, **f–h** and **j–l**

To investigate the neuroendocrine and autonomic components of the PVN, we characterized the Oxt-ir, AVP-ir and CRH-ir neurons. We observed a significant reduction in cell density, soma size and relative area of coverage of Oxt-ir neurons in the T2DM individuals, compared to the control group (Fig. 2a–d). Interestingly, the loss of Oxt-ir neurons was more severe in T2DM patients who were not treated with insulin (Additional file 2: Fig. S2 a-c). Although the cell density of AVP-ir neurons (Fig. 2e) was similar in T2DM and controls (Fig. 2f), the size of their soma and the relative area of coverage were smaller in the T2DM group (Fig. 1g, h). In contrast, we did not observe any changes in CRH-ir neurons in T2DM individuals (Fig. 2 i-l). Furthermore, insulin treatment did not affect AVP-ir or CRH-ir neuronal parameters as it did with the Oxt neurons (Additional file 2: Fig. S1d-i). Owing to these results, we evaluated malanocortinergic innervation into the PVN through alpha-MSH-ir fibers. We observed a significant reduction of alpha-MSH-ir fibers in the PVN (Additional file 2: Fig. S2a, b), indicating a defective melanocortinergic signalling pathway in the PVN of T2DM subjects. Taken together, our results suggest that a hypothalamic neuropeptidergic imbalance associated with T2DM pathophysiology occurs in the PVN, with a selective reduction of oxytocinergic neurons.

### Microglial activity in the PVN is unaltered in T2DM individuals

**Fig. 3:**
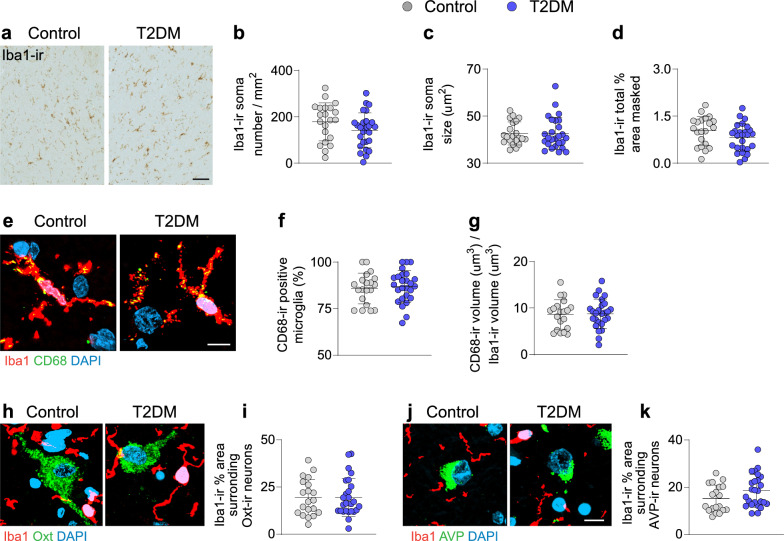
Microglia number and function are unaltered in the PVN of T2DM individuals. **a** Representative images of Iba1-ir in the PVN of control (n = 20) and T2DM (n = 26) subjects. Plot of microglial parameters **b** Iba1-ir soma number/mm^2^ (cell density), **c** Iba1-ir average soma size and **d** relative masked area by the Iba1-ir microglial cells. **e** Co-localization of CD68 (a phagosome indicator) and Iba1 in the PVN and **f** Quantitative analysis of CD68-ir positive microglia in percentage and **g** volume percentage of CD68-ir in relation to Iba1-ir. **h** Co-labelling of Oxt and Iba1 in the PVN and mean relative area masked (%) of Iba1-ir particles surrounding Oxt-ir neurons within 10 µm radius (**i**). **j** Co-labelling of AVP and Iba1 in the PVN and **k** mean relative area of coverage (%) of Iba1-ir particles surrounding AVP-ir neurons within 10 µm radius. Scale bar: 20 µm in **a**; 10 µm in **e** and 8 µm in **h** and **j**. Data are represented as mean ± SEM. *p < 0.05. Significance was calculated using Student`s t test in **b**–**d**, **f, g, i, k**

To investigate whether the Oxt-ir neuronal reduction was associated with increased microglial activity, we profiled Iba1-ir microglia in the PVN. Number, size and relative area of coverage of Iba1 + cells were comparable in both control and T2DM subjects in the Oxt-ir region (Fig. 3a–d). Comparably, we did not detect numeric changes in AVP-ir and CRH-ir covered areas (Additional file 2: Fig. S3). We also measured the phagocytic capacity using Iba1-ir and CD68-ir co-staining and found no differences in CD68-ir content between T2DM and controls, suggesting similar microglial immune surveillance and phagocytic capacity (Fig. 3e–g). Furthermore, insulin treatment in T2DM did not influence microglial abundance or phagocytic activity (Additional file 2: Fig. S4a-e).

We further investigated the relationship between microglial and neuronal parameters by linear regression analysis (Additional file 2: Figs. S5–S7). In T2DM subjects, we observed a positive correlation between Oxt-ir neurons soma size/total masked area and microglial density/ total area masked (Additional file 2: Fig. S5b, c, e, f). We also found a positive correlation between AVP-ir soma size and microglial cell density and total masked area in individuals with T2DM (Additional file 2: Fig. S6b, e). Lastly, CRH-ir soma size and total area were positively correlated to microglial total area (Additional file 2: Fig. S7e, f). However, no correlation was found in the control group, nor for the remaining comparisons in diabetic subjects (Additional file 2: Figs. S5–S7).

To investigate whether the observed changes in Oxt neurons were due to augmented neuron-microglia contacts, as previously reported between reactive microglia and proopiomelanocortin (POMC) neurons in mice with diet-induced obesity [58], we evaluated microglial presence surrounding Oxt-ir and AVP-ir neurons. We co-labelled Oxt or AVP with Iba1 and evaluated Iba1-ir within 10 µm radius from each individual neuron rendered. However, we found no differences in microglial proximity to Oxt-ir neurons (Fig. 3h–i) or AVP-ir neurons (Fig. 3j–k). Nevertheless, we observed increased microglial-occupation neighbouring Oxt neurons in T2DM subjects who were not undergoing insulin treatment prior to their demise in comparison to those with insulin treatment (Additional file 2: Fig. S4f). Microglial representation neighbouring AVP-ir neurons was unaltered regardless of treatment (Additional file 2: Fig. S4g).

### Reduced GFAP-ir astroglia and overrepresented glymphatic components in the PVN in T2DM

**Fig. 4:**
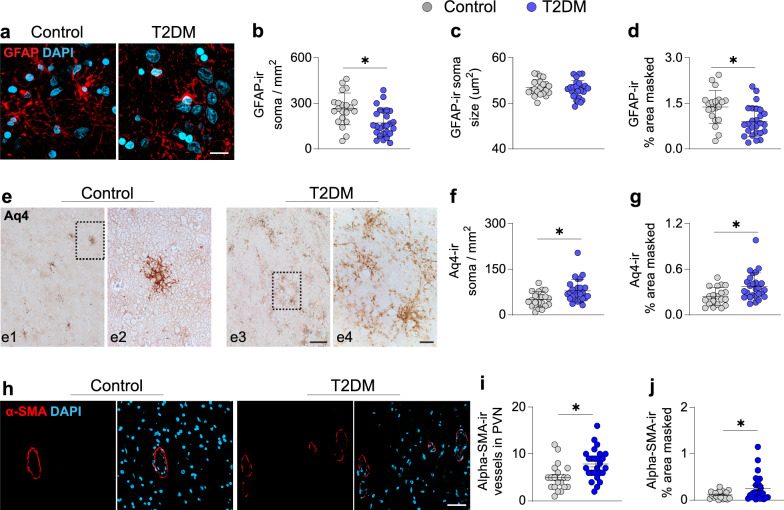
Loss of GFAP-ir astrocytes and overrepresentation of gliovascular components Aq4-ir astrocytes in the PVN of T2DM individuals. **a** Representative images of GFAP-ir in the PVN of control (n = 20) and T2DM (n = 26) subjects. Plot of astrocytic parameters **b** GFAP-ir soma number/mm^2^ (cell density), **c** GFAP-ir average soma size and **d** relative masked area by GFAP-ir astrocytes. **e** Representative images of Aq4-ir in the PVN of control and T2DM subjects, the cells framed in the left panel (**e1** and **e3**) are displayed at a high magnification in the right panel (**e2** and **e4**). **f** Quantitative analysis of Aq4-ir cells/mm^2^ (density) and **g** relative masked area of Aq4-ir astroglia. **h** Representative images of alpha-SMA-ir in the PVN of control and T2DM subjects and **i** plots of alpha-SMA-ir vessel number in the PVN; **j** quantitative analysis of alpha-SMA-ir masked area. Scale bar: 10 µm in **a, e2** and **e4,** 50 µm in **e1** and **e3**; 40 µm in **h**

Next, we assessed hypothalamic astrocytes in the region of Oxt-ir neurons (Fig. 4a). We found that GFAP-ir cell density and area of coverage were reduced compared to controls (Fig. 4b, d), while soma size remained comparable (Fig. 4c). Recent evidence suggested that a subpopulation of astrocytes characterized by Aq4, a water channel, is involved in the progression of neuropathology [31]. We evaluated Aq4-ir in the PVN (Fig. 4e) because Aq4 participation in hypothalamic dysfunction is virtually unexplored. Our findings showed an increased cell density and area of coverage of Aq4-ir astrocytes in the T2DM group (Fig. 4f, g), indicating a potential higher demand for drainage of unneeded cellular and metabolic wastes in the hypothalamic microenvironment. Furthermore, as Aq4 is an essential component of the glymphatic system [4], we also evaluated alpha-SMA, an endothelial marker for arteries and arterioles. We detected an increased area of coverage of alpha-SMA-ir in the PVN of T2DM subjects (Fig. 3h-j, Additional file 2: Fig. S8), indicating T2DM-associated angiogenesis also takes place in the PVN. Importantly, insulin treatment had no effect on any of these astrocytic or gliovascular markers (Additional file 2: Fig. S9). These results indicate that astrocytic dysfunction may contribute to T2DM pathophysiology, which is associated with hypervascularization in the PVN.

### Putative confounder analysis

We conducted a confounder analysis to account for potential influences from age, fixation time, postmortem delay, body mass index (BMI), post-absorptive glucose, and pH of cerebrospinal fluid (CSF). All of these factors were well-matched between the control and T2DM subjects. Although linear regression analysis showed some parameters to be incidentally significant, this did not affect the implications of our findings (Additional file 2: Figs. S10-S30 with statistics summarized in Additional file 1: Tables S4-S6).

## Discussion

In this study, we examined the neuronal and glial populations in the PVN of the hypothalamus in T2DM subjects and matched controls. Our analysis revealed a selective reduction in the density of PVN Oxt-ir neurons compared to other PVN neural populations in T2DM individuals. To investigate potential underlying mechanisms for the loss of oxytocinergic neurons we also profiled microglial and astroglial cells in the PVN. We found no changes in the microglia population, but our results showed a reduction in GFAP-ir astrocytic cells, while a subpopulation of Aq4-ir astrocytes was overrepresented in the PVN of T2DM individuals compared to the controls. These Aq4-ir astrocytes are important for the make-up of the glymphatic system. We also observed an increased presence of alpha-SMA in the PVN, indicating angiogenesis in the T2DM human brain. These findings suggest a disturbance of the Oxt neuron-astrocytes-vasculature system in the PVN of individuals with T2DM.

To gain more insight into the hypothalamic control of energy homeostasis, much research has focused on the POMC and neuropeptide Y / agouti-related peptidergic neurons in the Arc (IFN in human) [25, 33]. However, this is just one component of a complex and multi-layered control system that involves a range of neural networks and glia cells. The PVN acts as a central hub for metabolic control, as it receives and integrates afferent inputs from various intra- and extra-hypothalamic areas, such as the IFN, suprachiasmatic nucleus (SCN), and hindbrain [49]. Previous observations of loss of specific metabolism-controlling neurons in postmortem hypothalamic material suggest an imbalance of IFN [30] and SCN [24] neurons in T2DM, confirming changes in the human brain in this pathology [36]. However, despite its highly integrative role, the involvement of the PVN in diabetes progression has remained unexplored. Dysfunction in this nucleus is likely to be intimately associated with hyperglycemia and body weight fluctuations. Our study addresses this knowledge gap and highlights the importance of the PVN in T2DM pathology.

Oxt is a hypothalamic neuropeptide that regulates a broad range of physiological processes, including social cognition and energy homeostasis [8, 11, 23]. It is produced in the hypothalamic PVN and supraoptic nucleus [11, 23] and its effects are exerted throughout the central nervous system and periphery [45, 51]. Oxt receptor expression is observed in key metabolic organs such as adipose tissue [51] and endocrine pancreas [39], indicating its role in regulating adiposity and pancreatic function. Oxt-knockout mice develop obesity and glucose intolerance, highlighting the importance of Oxt in these processes [12]. Recent research suggests that PVN Oxt neurons respond to glucoprivation, and are necessary for the pancreatic beta cells response to glucose fluctuations [40]. Although Oxt is well-known for its anorexic effects [37], direct manipulation of PVN Oxt neurons is not sufficient to affect food consumption or body weight [6, 47, 52]. Instead, neuroanatomical, and functional circuit mapping of feeding behaviour suggests that the participation of PVN Oxt neurons in feeding is dependent on synaptic inputs from the Arc (IFN in human) [6].

Interestingly, in normal weighted individuals, serum Oxt levels decrease during fasting and correlate negatively with various metabolic parameters, such as the BMI, fasting and postprandial glucose/insulin concentrations, and insulin sensitivity [42]. However, Oxt levels do not differ between glucose-tolerant overweight and lean subjects [42]. In T2DM individuals, serum Oxt levels are lower than those in nondiabetic individuals, irrespective of obesity [2, 42]. These findings suggest that glycaemic control is a central mediator of oxytocin’s influence on metabolic homeostasis. Whether reductions in serum Oxt levels in T2DM are due to defective hypothalamic neuroendocrine systems was undefined. Oxt has been considered as a therapeutic approach to T2DM, as it has been shown to lower glycaemic levels in mice and humans [18]. Our study reports a reduction in Oxt neurons in the PVN of T2DM. Notably, especially the absence of antidiabetic treatment (insulin) was accompanied by lesser Oxt-ir neurons, indicating greater hypothalamic dysfunction in poorly controlled T2DM, as suggested previously [30]. It is noteworthy that selectively Oxt-ir neurons were reduced in PVN of T2DM individuals, indicating a greater vulnerability of this neuronal population to metabolic stressors. Our findings suggest a central role for Oxt neurons in glucoregulation, with potential implications in the development and progression of T2DM.

Glia malfunctioning is considered a critical mechanistic node in hypothalamic dysfunction in metabolic disorders [29]. Our data showed the loss of Oxt neurons is associated with a reduction in GFAP-positive astrocytes. Similarly, GFAP-ir astrocytes were also reduced in the SCN of T2DM subjects, alongside the loss of SCN AVP- and vasoactive intestinal peptide-containing neurons [24]. These results suggest that this sub-population of astrocytes might play a vital role in maintaining local homeostasis for selective populations of neurons in different hypothalamic regions. In recent years the essential role of the glymphatic system, which aids in removing waste products from the central nervous system, has become clear [28, 50, 59]. Aq4-expressing astroglia contribute to this process by selectively permitting water diffusion and maintaining ionic and osmotic homeostasis [4]. Recent studies have suggested that changes in Aq4 expression may be linked to restrictions in CSF flow and the accumulation of waste products, resulting in neuronal dysfunction and cognitive decline [26]. This idea is supported by the observation that Aq4-knockout animals show defective removal of amyloid beta plaques [26, 50]. Moreover, an increased number of Aq4-astrocytes has been observed in the frontal cortex of aged and cognitive impaired subjects, further reinforcing their importance in draining brain wastes [59]. Interestingly, T2DM patients have elevated protein levels in the CSF [32]. This could potentially be associated with an enhanced glymphatic clearance activity. To investigate this hypothesis, techniques such as magnetic resonance imaging (MRI) have proven valuable in identifying glymphatic flow and clearance rate in humans [1, 44, 61].

The role of Aq4 in the hypothalamus is still underexplored, although a recent study suggested increased activity of the glymphatic system in the hypothalamus in long-term high fat-fed mice [17]. Our findings regarding the increased numbers of Aq4-ir astrocytes in the hypothalamus of T2DM subjects may indicate an elevated need for microenvironmental clearance through the glymphatic system. This increased demand could be due to neuronal injury in the PVN, as evidenced by the loss of Oxt neurons. It could also be induced by T2DM-associated insulin resistance, as it is known that the suppression of insulin signalling in astrocytes leads to increased angiogenesis and altered blood flow [19]. Therefore, similar to what has been suggested for rodents on a long-term obesogenic diet [17], T2DM-associated insulin resistance may play a role in increased fluid trafficking in the hypothalamus in an Aq4-dependent manner. Another T2DM-associated pathological change that may contribute to the increased demand for clearance are disrupted circadian rhythms and impaired sleep quality, as adequate sleep quality ensures a sufficient removal of potentially neurotoxic waste products that accumulate in the awake central nervous system [55]. This idea is based on the fact that in the SCN, where the brain's master circadian clock is located, arginine vasopressin- and vasoactive intestinal polypeptide-containing neurons and astroglial cells, which are critical in maintaining the circadian clock, were significantly reduced in individuals with T2DM [24]. These findings also indicate that improving sleep quality for T2DM patients is expected to not only enhance waste clearance but also prevent the onset of AD, which is known to be highly associated with T2DM.

Microglia are the resident immune cells of the central nervous system, and in diet-induced obesity, they are known to be activated into a proinflammatory state [9, 16, 21, 22]. Although the role of these cells in T2DM is not yet well understood, previous studies did not find changes in microglia number in the IFN and SCN of T2DM subjects [24, 30], suggesting no or a milder change in microglial profile in T2DM compared to obesity. Our current findings support these previous observations, as we did not observe any morphological or functional changes in microglia in the PVN of T2DM. Alterations in neuronal soma size are closely associated with neuronal stress and have been reported in various neuropathology studies [5, 15, 54]. Interestingly, in our study, we found a positive correlation between microglial density and PVN neuropeptide soma size (and consequently, total area of coverage) exclusively in T2DM subjects. This suggests that microglia might be more active in T2DM and contribute to hypothalamic neuronal dysfunction. Furthermore, emerging evidence indicates that distinct subpopulations of microglia are associated with the progression and development of diseases in the human brain [41, 48]. Future studies that profile microglial interactions with specific neuronal populations at a single-cell level are needed to determine whether specific and localized microglial populations are linked to neurological outcomes in T2DM. It is also worth noting that microglia appear to be sensitive to antidiabetic treatments, as we found that patients who were not undergoing insulin treatment had a higher microglial representation neighbouring Oxt neurons. These findings suggest that microglial activity in response to T2DM-related stressors may contribute to the observed changes in neuronal parameters. However, more research is needed to fully understand the underlying mechanisms and implications of these correlations.

Taken together with our findings on astrocytes, these results raise fundamental questions on how the human brain partitions the innate immune microglia-governed phagolysosome cleaning system and the glymphatic draining system to maintain a healthy brain microenvironment that ensures neuronal survival. It is possible that human microglia are more resilient to immune challenges from the microenvironment than rodents, as we hardly detected changes in microglia in T2DM. In contrast, the glymphatic system might be more sensitive to T2DM-associated pathological changes, as evidenced by the increased number of Aq4-expressing astrocytes. Further research is needed to fully understand the mechanisms underlying these observations.

## Supplementary Information

**Additional file 1.** List of antibodies for immunohistochemestry and immunofluorescence and detailed statistical information.

**Additional file 2.** Supplementary information and correalation analysis between neuronal and glial parameters with potential confounders.

## Data Availability

All data generated or analyzed during this study are included in this published article (and its Additional files 1 and 2).
