# Supplementary material for: Reduction of oxytocin-containing neurons and enhanced glymphatic activity in the hypothalamic paraventricular nucleus of patients with type 2 diabetes mellitus
Source: Acta Neuropathol Commun. 2023 Jul 3;11:107. doi: 10.1186/s40478-023-01606-w (PMC10318717; doi:10.1186/s40478-023-01606-w)
Supplement: Supplementary file 1 — Additional file 1. List of antibodies for immunohistochemestry and immunofluorescence and detailed statistical information. [file 40478_2023_1606_MOESM1_ESM.pdf]

Supplementary Table 1: Antibody information

Primary antibody	Source	Host	Catalog number	Specificity (PMID)	Diluition
AVP (IHC)	Netherlands Institute for Brain Ressarch	Rabbit (Truss86)	C.P.230686	3243229	1 / 1000
AVP (IF)	Netherlands Institute for Brain Ressarch a gift from dr. F.W. Leuween	Mouse	III-D-7; RRID:AB_2313980	7723930, 17072839, 7819342	1 / 300
Oxytocin-neurophysin 1 (IHC)	Abcam	Rabbit	ab2078	28415720	1 / 10000
Oxytocin (IF)	Netherlands Institute for Brain Ressarch a gift from dr. F.W. Leuween	Mouse	OT-A-I-28	28447621	1 / 1000
alpha-MSH	Millipore	Sheep	AB5087	28591639 (mice or human)	1 / 5000
CRH	Netherlands Institute for Brain Ressarch	Rat	PFU83	17308368	1 / 100000
Iba1	Synaptic Systems	Rabbit	234003	32814716	1 / 200
CD68	Agilent	Mouse	M0814 (clone KP1)	36497449	1 / 400
GFAP	DAKO	Rabbit	Z0334	32422642	1 / 1000
Aquaporin 4	Atlas antibodies	Rabbit	HPA014784	27893874	1 / 1000
alpha-SMA	Sigma Aldrich	Mouse	A5228	24024123	1 / 1000

Supplementary Table 2: Mean values (± standard deviation) and associated p-values in this manuscript

Parameter	Control	T2DM	p-value
Oxt-ir soma number / mm²	91.40 ± 22.23	71.50 ± 18.01	**0.001**
Oxt-ir soma size (µm²)	115.70 ± 9.50	107.10 ± 15.89	**0.039**
Oxt-ir (% area masked)	1.062 ± 0.281	0.779 ± 0.250	< **0.001**
AVP-ir soma number / mm²	110.70 ± 27.35	100.9 ± 30.760	0.269
AVP-ir soma size (µm²)	109.80 ± 16.65	98.30 ± 15.42	**0.017**
AVP-ir (% area masked)	1.235 ± 0.399	0.999 ± 0.362	**0.042**
CRH-ir soma number / mm²	56.23 ± 31.70	58.99 ± 37.20	0.792
CRH-ir soma size (µm²)	66.29 ±13.06	65.96 ± 13.84	0.935
CRH-ir (% area masked)	0.388 ± 0.239	0.435 ± 0.306	0.576
alpha-MSH-ir (% area masked)	0.347 ± 0.256	0.156 ± 0.086	**0.001**
Iba1-ir soma number / mm² (Oxt-ir area)	178.10 ± 81.95	141.70 ± 75.28	0.120
Iba1-ir soma size (µm²) (Oxt-ir area)	42.64 ± 4.86	42.65 ± 6.77	0.997
Iba1-ir (% area masked) (Oxt-ir area)	1.031 ± 0.459	0.817 ± 0.434	0.108
Iba1-ir soma number / mm² AVP-ir area)	182.40 ± 86.25	143.30 ± 75.43	0.103
Iba1-ir soma size (µm²) AVP-ir area)	42.51 ± 4.796	42.40 ± 5.934	0.945
Iba1-ir (% area masked) AVP-ir area)	1.052 ± 0.481	0.803 ± 0.435	0.069
Iba1-ir soma number / mm² CRH-ir area)	157.50 ± 66.79	127.6 ± 62.81	0.121
Iba1-ir soma size (µm²) CRH-ir area)	42.19 ± 4.54	40.91 ± 5.63	0.402
Iba1-ir (% area masked) CRH-ir area)	0.912 ± 0.372	0.750 ± 0.421	0.173
CD68-ir positive microglia (%)	85.79 ± 8.27	86.79 ± 8.85	0.639
CD68-ir volume µm^3	8.61 ± 3.17	8.77 ± 3.14	0.867
Iba-ir % area surrounding Oxt-ir neurons	19.41 ± 5.74	19.46 ± 6.90	0.986
Iba-ir % area surrounding AVP-ir neurons	15.12 ± 9.52	18.47 ± 10.10	0.094
GFAP-ir soma number / mm²	263.10 ± 105.23	170.30 ± 91.04	**0.002**
GFAP-ir soma size (µm²)	53.48 ± 1.75	52.99 ± 1.97	0.388
GFAP-ir (% area masked)	1.373 ± 0.548	0.903 ± 0.489	**0.003**
Aq4-ir soma number / mm²	50.41 ± 25.30	79.35 ± 37.33	**0.004**
Aq4-ir (% area masked)	0.241 ± 0.117	0.374 ± 0.179	**0.006**
alpha-SMA-ir (% area masked)	0.114 ± 0.079	0.256 ± 0.278	**0.032**
alpha-SMA-ir vessels in the PVN	5.05 ± 2.855	3.196 ± 0.079	**0.036**

Supplementary Table 3: Mean values (± standard deviation) and associated p-values of parameters in T2DM with or without insulin treatment

Parameter	T2DM - insulin	T2DM + insulin	p-value
Oxt-ir soma number / mm²	80.09 ± 12.14	64.3 ± 19.30	**0.020**
Oxt-ir soma size (µm²)	110.5 ±14.48	104.2 ± 11.68	0.322
Oxt-ir (% area masked)	0.888 ± 0.174	0.685 ± 0.271	0.361
AVP-ir soma number / mm²	94.11 ± 32.48	106.8 ± 29.09	0.304
AVP-ir soma size (µm²)	96.67 ± 17.90	99.69 ± 13.48	0.627
AVP-ir (% area masked)	0.999 ±0.386	1.067 ± 0.340	0.311
CRH-ir soma number / mm²	51.88 ± 26.90	70.58 ± 37.24	0.161
CRH-ir soma size (µm²)	64.74 ±13.63	67.00 ± 14.45	0.686
CRH-ir (% area masked)	0.364 ± 0.280	0.496 ± 0.325	0.364
Iba1-ir soma number / mm² (Oxt-ir area)	133.00 ± 71.12	149.20 ± 80.56	0.596
Iba1-ir (% area masked) (Oxt-ir area)	0.588 ± 0.330	0.603 ± 0.344	0.912
CD68-ir positive microglia (%)	86.69 ± 7.88	87.72 ± 9.23	0.555
CD68-ir volume μm^3	8.40 ± 3.76	9.19 ± 2.57	0.532
Iba-ir % area surrounding Oxt-ir neurons	15.02 ± 7.33	23.26 ± 10.82	**0.035**
Iba-ir % area surrounding AVP-ir neurons	18.83 ± 8.44	18.15 ± 5.44	0.815
GFAP-ir soma number / mm²	154.20 ± 101.61	184.00 ± 82.22	0.415
GFAP-ir soma size (µm²)	52.78 ± 2.245	53.18 ± 1.781	0.619
GFAP-ir (% area masked)	0.813 ± 0.537	0.981 ± 0.449	0.392
Aq4-ir soma number / mm²	90.51 ± 46.35	69.79 ± 25.45	0.162
Aq4-ir (% area masked)	0.429 ± 0.224	0.326 ± 0.120	0.148
alpha-SMA-ir (% area masked)	0.224 ± 0.249	0,282 ± 0.309	0.607

Supplementary Table 4: Linear regression of hypothalamic neuropeptides parameters in relation to potential co-founders

Variable	Control		T2DM	
	**R^2**	**p-value**	**R^2**	**p-value**
Oxt-ir soma number / mm^2				
Age (years)	0.1132	0.1469	0.0143	0.5604
Postmortem delay (hours)	0.0018	0.8578	0.0020	0.8260
Fixation time (days)	0.0171	0.6422	0.0288	0.4383
BMI	0.0577	0.3073	0.4060	0.0649
Cerebrospinal fluid pH	0.0552	0.4187	0.0682	0.2403
Postabsorvative glucose	0.5354	0.0616	0.0060	0.8000
Oxt-ir soma size (μm^2)				
Age (years)	< 0.0001	0.9669	0.0297	0.3994
Postmortem delay (hours)	0.0830	0.2178	0.1189	0.0853
Fixation time (days)	0.2535	0.0557	0.0014	0.8631
BMI	0.0034	0.8064	0.6022	**0.0140**
Cerebrospinal fluid pH	0.0888	0.3007	0.3261	**0.0051**
Postabsorvative glucose	0.5641	0.0517	0.3080	**0.0490**
Oxt-ir (% area masked)				
Age (years)	0.0840	0.2150	0.0037	0.7662
Postmortem delay (hours)	0.0143	0.6265	0.0086	0.6513
Fixation time (days)	0.0001	0.9658	0.0142	0.5869
BMI	0.0312	0.4557	0.6403	**0.0096**
Cerebrospinal fluid pH	0.0820	0.3207	0.1852	**0.0456**
Postabsorvative glucose	0.6138	**0.0371**	0.1146	0.2580
AVP-ir soma number / mm^2				
Age (years)	0.0561	0.3146	0.0415	0.3182
Postmortem delay (hours)	0.0629	0.2858	0.1267	0.0743
Fixation time (days)	0.0812	0.3032	0.0193	0.5267
BMI	0.0029	0.8197	0.0054	0.8508
Cerebrospinal fluid pH	0.0744	0.3453	0.0152	0.5842
Postabsorvative glucose	0.0577	**0.0476**	0.0009	0.9211
AVP-ir soma size (μm^2)				
Age (years)	0.1042	0.1651	0.1267	0.0836
Postmortem delay (hours)	0.0587	0.3032	0.1267	0.0836
Fixation time (days)	0.0440	0.4592	0.0239	0.4809
BMI	< 0.0001	0.9911	0.0756	0.4738
Cerebrospinal fluid pH	0.0097	0.7368	0.0077	0.6969
Postabsorvative glucose	0.0577	**0.0476**	0.0009	0.9211
AVP-ir (% area masked)				
Age (years)	0.0067	0.7312	0.1174	0.0866
Postmortem delay (hours)	0.0041	0.7878	0.0423	0.3130
Fixation time (days)	0.0288	0.5354	0.0052	0.7419
BMI	0.0011	0.8848	0.0422	0.5959
Cerebrospinal fluid pH	0.0703	0.3594	0.0196	0.5333
Postabsorvative glucose	0.7745	**0.0090**	0.0560	0.4363
CRH-ir soma number / mm2				
Age (years)	0.1429	0.1003	0.0897	0.1371

Postmortem delay (hours)	0.0083	0.7016	0.0840	0.1508
Fixation time (days)	0.2148	0.0819	0.0021	0.8329
BMI	0.1355	0.1165	0.0166	0.7411
Cerebrospinal fluid pH	0.0007	0.9205	0.0027	0.8153
Postabsorvative glucose	0.4693	0.0894	0.1526	0.1869
CRH-ir soma size (μm²)				
Age (years)	0.0467	0.3465	0.0414	0.3185
Postmortem delay (hours)	< 0.0001	0.9985	0.2334	**0.0124**
Fixation time (days)	0.0059	0.7759	0.0003	0.9858
BMI	0.0896	0.2599	0.1192	0.4484
Cerebrospinal fluid pH	0.0410	0.3784	0.0431	0.5918
Postabsorvative glucose	0.2958	0.2069	0.2178	0.1079
CRH-ir (% area masked)				
Age (years)	0.0890	0.1888	0.0640	0.2124
Postmortem delay (hours)	0.1190	0.6379	0.1677	**0.0378**
Fixation time (days)	0.1187	0.1912	0.0001	0.9590
BMI	0.1410	0.0934	0.0070	0.8296
Cerebrospinal fluid pH	0.0012	0.9046	0.0174	0.5579
Postabsorvative glucose	0.5021	0.0747	0.2436	0.0865
alpha-MSH-ir (% area masked)				
Age (years)	0.0356	0.4255	0.0001	0.9605
Postmortem delay (hours)	0.0475	0.3557	0.1204	0.0824
Fixation time (days)	0.0302	0.5353	0.0479	0.3154
BMI	0.0768	0.2366	0.0408	0.6019
Cerebrospinal fluid pH	0.0135	0.6923	0.0111	0.6396
Postabsorvative glucose	0.3927	0.0938	0.1138	0.2597

Supplementary Table 5: Linear regression of microglial parameters in relation to potential co-founders

Variable	Control R^2	Control p-value	T2DM R^2	T2DM p-value
Iba1-ir soma number / mm²				
Age (years)	0.0026	0.8284	0.0074	0.6755
Postmortem delay (hours)	0.0151	0.6049	0.0105	0.6172
Fixation time (days)	0.0786	0.3112	0.0584	0.2666
BMI	0.0631	0.2854	0.0372	0.6188
Cerebrospinal fluid pH	0.0001	0.9660	0.1741	0.0534
Postabsorvative glucose	0.5756	**0.0480**	0.3978	**0.0208**
Iba1-ir soma size (µm²)				
Age (years)	0.0326	0.4461	0.0249	0.4405
Postmortem delay (hours)	0.0089	0.6919	0.1181	0.0856
Fixation time (days)	0.1226	0.2007	0.0203	0.5157
BMI	0.0004	0.9288	0.3685	0.0830
Cerebrospinal fluid pH	0.0018	0.8851	0.0215	0.5146
Postabsorvative glucose	0.3002	0.2029	0.0832	0.3390
Iba1-ir (% area masked)				
Age (years)	0.0026	0.8288	0.0227	0.4617
Postmortem delay (hours)	0.1020	0.6718	0.0297	0.3996
Fixation time (days)	0.0690	0.3440	0.0610	0.2527
BMI	0.4710	0.3579	0.0404	0.6037
Cerebrospinal fluid pH	0.0005	0.9387	0.1776	0.0507
Postabsorvative glucose	0.5054	0.0733	0.4238	**0.0160**
CD68-ir volume µm3 / Iba1-ir volume microglia µm3				
Age (years)	0.1099	0.1533	0.0038	0.7640
Postmortem delay (hours)	0.0016	0.9575	0.0006	0.8995
Fixation time (days)	0.0028	0.8507	0.0106	0.6401
BMI	0.0239	0.5145	0.0416	0.5790
Cerebrospinal fluid pH	0.0849	0.3120	0.1775	0.0508
Postabsorvative glucose	0.1397	0.4089	0.0272	0.5899
Iba-ir % area surrounding Oxt-ir neurons				
Age (years)	0.1937	0.0521	0.0569	0.2403
Postmortem delay (hours)	0.0051	0.7636	0.1100	0.6092
Fixation time (days)	0.0270	0.5580	0.0008	0.8923
BMI	0.0031	0.8063	0.1268	0.3469
Cerebrospinal fluid pH	0.1555	0.1629	0.1310	0.1069
Postabsorvative glucose	0.1389	0.4102	0.2142	0.1112
Iba-ir % area surrounding AVP-ir neurons				
Age (years)	0.2143	**0.0460**	0.0101	0.6330
Postmortem delay (hours)	0.0531	0.3423	0.0803	0.1699
Fixation time (days)	0.0032	0.8475	0.0349	0.4051
BMI	0.1600	0.0898	0.0462	0.5786
Cerebrospinal fluid pH	0.2546	0.0787	0.0036	0.8013
Postabsorvative glucose	0.4924	0.1202	0.3624	**0.0384**

Supplementary Table 6: Linear regression of astrocytic parameters in relation to potential co-founders

Variable	Control R^2	Control p-value	T2DM R^2	T2DM p-value
GFAP-ir soma number / mm²				
Age (years)	0.0012	0.8824	0.0810	0.1586
Postmortem delay (hours)	0.0967	0.1819	0.0274	0.4183
Fixation time (days)	0.0750	0.3232	0.0002	0.9440
BMI	0.0578	0.3082	0.0476	0.5725
Cerebrospinal fluid pH	0.0440	0.4716	0.1926	**0.0411**
Postabsorvative glucose	0.1030	0.8281	0.0020	0.8842
GFAP-ir soma size (μm²)				
Age (years)	0.0098	0.6776	0.0927	0.1303
Postmortem delay (hours)	0.0448	0.3700	0.0120	0.5930
Fixation time (days)	0.3084	0.7448	0.0051	0.7453
BMI	0.0123	0.6413	0.1725	0.2663
Cerebrospinal fluid pH	0.0016	0.8902	0.0907	0.1730
Postabsorvative glucose	0.5065	0.0729	0.1948	0.1311
GFAP-ir (% area masked)				
Age (years)	0.0017	0.5816	0.0761	0.1742
Postmortem delay (hours)	0.0832	0.2172	0.0280	0.4091
Fixation time (days)	0.0713	0.3357	< 0.0001	0.9669
BMI	0.0576	0.3077	0.6604	0.5237
Cerebrospinal fluid pH	0.0428	0.4776	0.2081	**0.0328**
Postabsorvative glucose	0.0202	0.7607	0.0009	0.9213
Aq4-ir soma number / mm²				
Age (years)	0.0048	0.7704	0.0091	0.6414
Postmortem delay (hours)	0.0005	0.9195	0.0308	0.3905
Fixation time (days)	0.0355	0.5012	0.0028	0.8082
BMI	0.0501	0.3425	0.0665	0.5029
Cerebrospinal fluid pH	0.0002	0.9601	< 0.0001	0.9691
Postabsorvative glucose	0.0244	0.7378	0.1048	0.2807
Aq4-ir (% area masked)				
Age (years)	0.0007	0.1212	0.0089	0.1827
Postmortem delay (hours)	0.0011	0.8855	0.0287	0.4076
Fixation time (days)	0.0233	0.5871	0.0003	0.9300
BMI	0.0712	0.2553	0.1093	0.3848
Cerebrospinal fluid pH	0.0014	0.8968	0.0015	0.8627
Postabsorvative glucose	0.0266	0.7266	0.1016	0.2885
alpha-SMA-ir (% area masked)				
Age (years)	0.0950	0.2133	0.1217	0.0807
Postmortem delay (hours)	0.0797	0.2561	< 0.0001	0.9680
Fixation time (days)	0.1893	0.1373	0.0722	0.1992
BMI	0.1610	0.0989	0.0044	0.8645
Cerebrospinal fluid pH	0.0004	0.9494	0.0011	0.8819
Postabsorvative glucose	0.0300	0.7424	< 0.0001	0.9910
alpha-SMA-ir (% area masked)				
Age (years)	0.0356	0.4255	0.0001	0.9605
Postmortem delay (hours)	0.0475	0.3557	0.1204	0.0824

Fixation time (days)	0.0302	0.5353	0.0479	0.3154
BMI	0.0768	0.2366	0.0408	0.6019
Cerebrospinal fluid pH	0.0135	0.6923	0.0111	0.6396
