# Supplementary material for: Reduction of oxytocin-containing neurons and enhanced glymphatic activity in the hypothalamic paraventricular nucleus of patients with type 2 diabetes mellitus
Source: Acta Neuropathol Commun. 2023 Jul 3;11:107. doi: 10.1186/s40478-023-01606-w (PMC10318717; doi:10.1186/s40478-023-01606-w)
Supplement: Supplementary file 2 — Additional file 2. Supplementary information and correalation analysis between neuronal and glial parameters with potential confounders. [file 40478_2023_1606_MOESM2_ESM.pdf]

Reduction of oxytocin-containing neurons and enhanced glymphatic activity in the hypothalamic paraventricular nucleus of patients with type 2 diabetes mellitus

Felipe Correa-da-Silva[1,2], Martin J. Kalsbeek[1,2], Femke S. Gadella[1], Jorn Oppersma[1], Wei Jiang[1,2], Samantha E.C. Wolff[1], Nikita L. Korpel[3], Dick F. Swaab[2], Eric Fliers[1], Andries Kalsbeek[1,2,3], Chun-Xia Yi[1,2,3]

Electronic Supplementary Materials

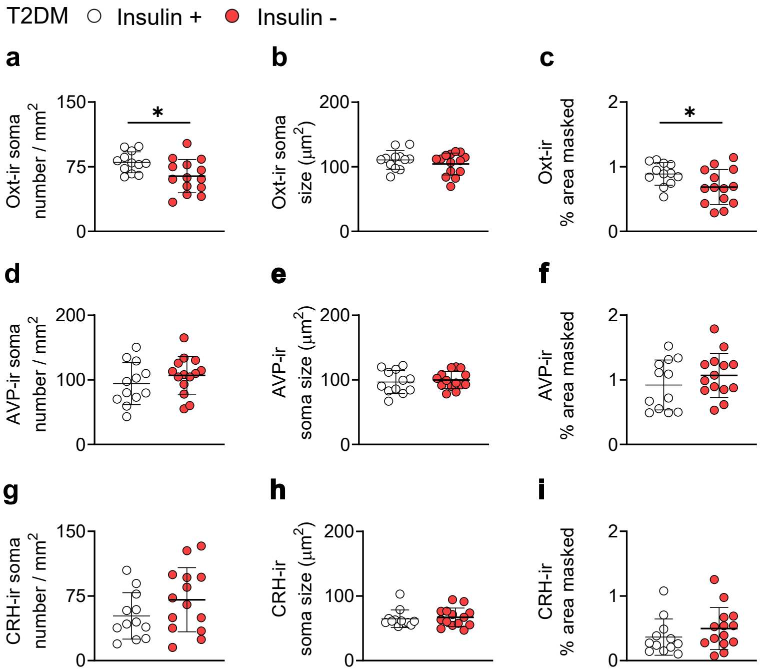

Supplementary Fig. 1

The antidiabetic treatment-associated effect on PVN neuronal populations. Comparison between T2DM individuals with or without insulin treatment. (**a**) Oxt-ir soma number / mm^2 (neuronal density), (**b**) Oxt-ir average soma size and (**c**) relative masked area by positive Oxt-ir cells. (**d**) Quantitative analysis of (**f**) AVP-ir soma number / mm^2 (neuronal density), (**e**) AVP-ir average soma size and (**f**) relative masked area by positive AVP-ir cells. (**g**) CRH-ir soma number / mm2 (neuronal density), (**h**) CRH-ir average soma size and (**i**) relative masked area by positive CRH-ir particles. Note that Oxt-ir neurons density and relative masked area are reduced in untreated T2DM individuals. AVP = arginine-vasopressin; CRH= corticotrophin releasing hormone; ir = immunoreactivity; PVN = paraventricular nucleus; T2DM = type 2 diabetic mellitus. Data are represented as mean ± SEM. * $p < 0.05$. Significance was calculated using Student`s t test in **a-i.** T2DM insulin + : n = 12, T2DM insulin – : n=14.

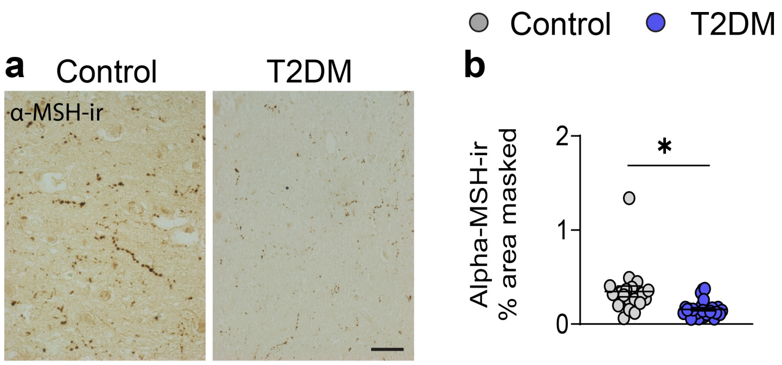

Supplementary Fig. 2

Decreased alpha-MSH fibers in the PVN of T2DM subjects. (**a**) Representative images of alpha-MSH-ir fibers in the PVN of control and type 2 diabetes diabetic subjects; and (**b**) plot of relative area masked of alpha-MSH-ir positive particles. Note that alpha-MSH fibers are reduced in the PVN. alpha-MSH = alpha-melanocortin stimulating hormone; PVN = paraventricular nucleus; ir = immunoreactivity; T2DM = type 2 diabetic mellitus. Scale bar 20 µm in **a**; and 20 µm in **e**. Data are represented as mean ± SEM. * $p < 0.05$. Significance was calculated using Student`s t test in **b**. Control: n = 20, T2DM: n = 26.

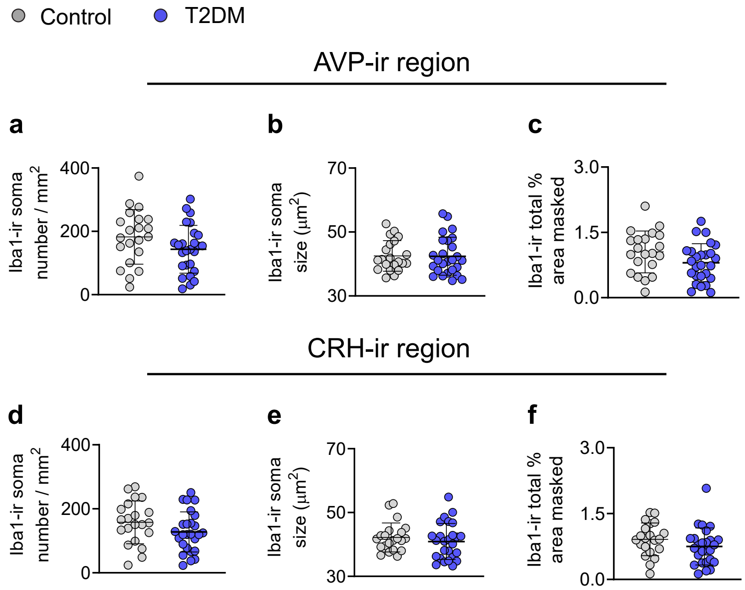

Supplementary Fig. 3

Microglia number is unchanged in the PVN of T2DM individuals. Quantitative analysis of Iba1-ir in the PVN (AVP-ir region) of control and T2DM subjects. Plot of microglial parameters (**a**) Iba1-ir soma number / mm^2 (cell density), (**b**) Iba1-ir average soma size and (**c**) relative masked area by positive microglial cells. Quantitative analysis of Iba1-ir in the PVN (CRH-ir region) of control and T2DM subjects. Plot of microglial parameters (**d**) Iba1-ir soma number / mm^2 (cell density), (**e**) Iba1-ir average soma size and (**f**) relative masked area of positive microglial cells. AVP = arginine-vasopressin; CRH= corticotrophin releasing hormone; ir = immunoreactivity; PVN = paraventricular nucleus; Iba1 = ionized calcium-binding adapter molecule 1; T2DM = type 2 diabetic mellitus. Data are represented as mean ± SEM. Control: n = 20, T2DM: n = 26.

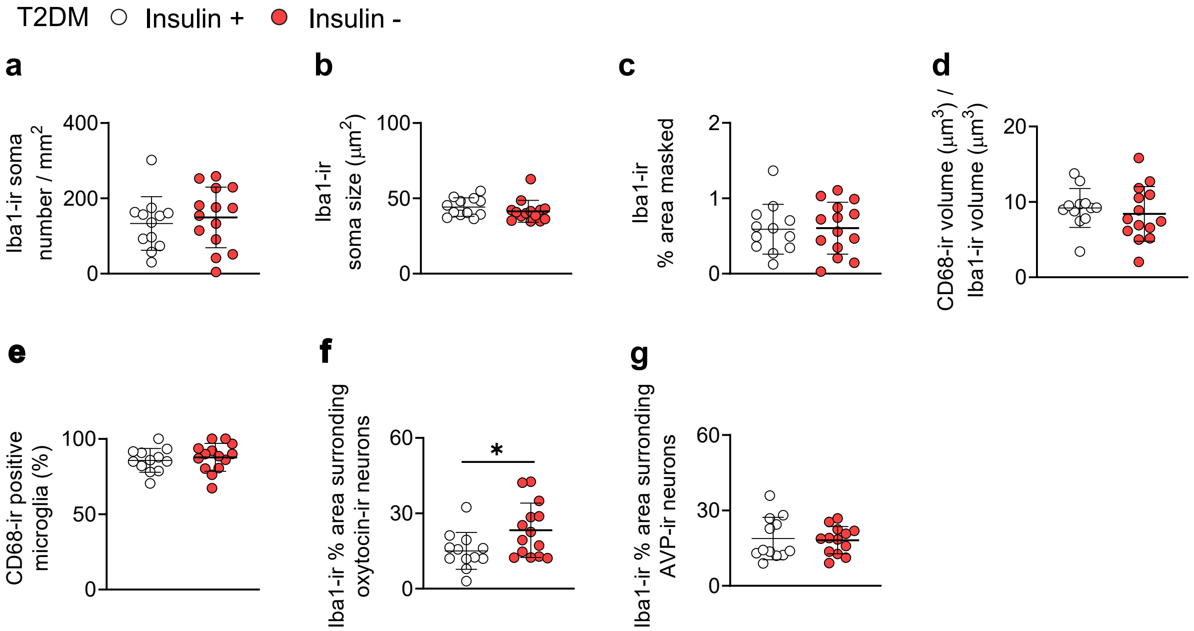

Supplementary Fig. 4

The antidiabetic treatment-associated effect on PVN microglia. Comparison between T2DM individuals with or without insulin treatment. (**a**) Iba1-ir soma number / mm^2 (cell density), (**b**) Iba1-ir average soma size and (**c**) relative masked area by positive microglial cells. (**d**) Volume percentage of CD68-ir in relation to Iba1-ir and (**e**) Quantitative analysis of CD68-ir positive microglia in percentage. Mean relative area of masked (%) of Iba1-ir particles surrounding Oxt-ir neurons (**f**) and AVP-ir neurons (**g**) within 10 µm. Please note that microglial features are unaltered in the PVN irrespective of insulin treatment, except for Iba1-ir particles abundance around Oxt neurons. These data suggest closer microglial contact in T2DM untreated subjects. Oxt = oxytocin; AVP = arginine-vasopressin; ir = immunoreactivity; PVN = paraventricular nucleus; Iba1 = ionized calcium-binding adapter molecule 1; CD68 = cluster of differentiation 68; T2DM = type 2 diabetic mellitus. Data are represented as mean ± SEM. * $p < 0.05$. Significance was calculated using Student`s t test in **a-g**, T2DM insulin + : n = 12, T2DM insulin – : n=14.

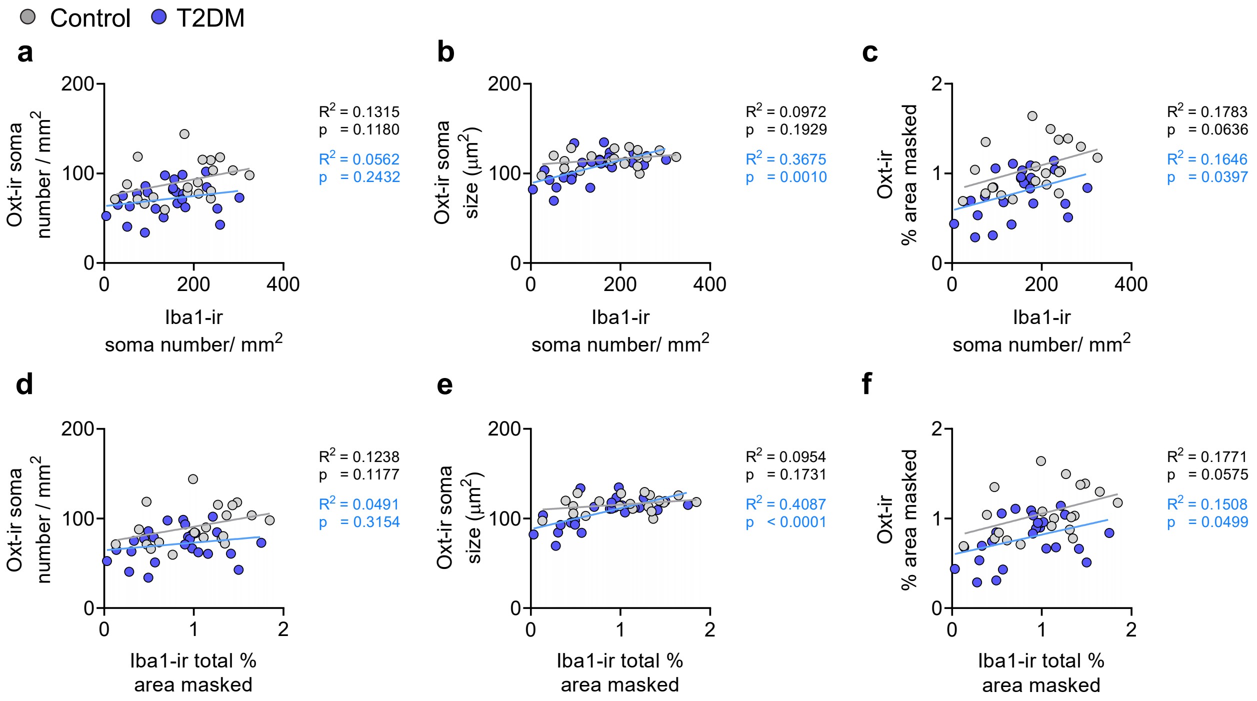

Supplementary Fig. 5

Analysis of oxytocin-ir (Oxt-ir) neurons in relation to microglial (Iba1-ir) cells in the PVN of control and T2DM subjects. Plots of (**a**) Oxt-ir neurons density in relation to Iba-ir microglial density; (**b**) Oxt-ir neurons soma size in relation to Iba1-ir microglial density and (**c**) relative masked area by Oxt-ir cells in relation to Iba1-ir microglial density. Plots of (**d**) Oxt-ir neurons density in relation to relative masked area of Iba1-ir microglial cells; (**e**) Oxt-ir neurons soma size in relation to relative masked area of Iba1-ir microglial cells (**f**) Oxt-ir relative masked area in relation to elative masked area of Iba1-ir microglial cells Oxt = oxytocin; Iba1 = ionized calcium-binding adapter molecule 1; PVN = paraventricular nucleus; ir = immunoreactivity; CSF = cerebrospinal fluid; post-abs=post absorptive; T2DM = type 2 diabetic mellitus. Control: n = 20, T2DM: n = 26.

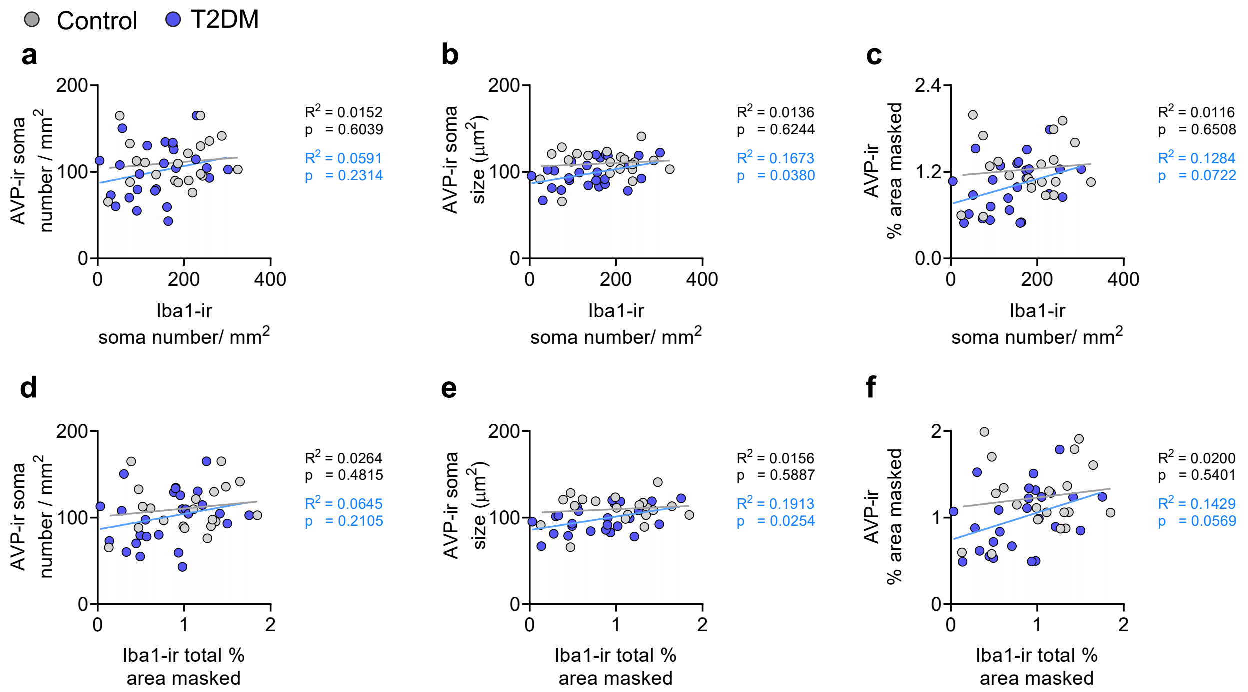

Supplementary Fig. 6

Analysis of AVP-ir (AVP-ir) neurons in relation to microglial (Iba1-ir) cells in the PVN of the hypothalamus of control and T2DM subjects. Plots of (**a**) AVP-ir neurons density in relation to Iba-ir microglial density; (**b**) AVP-ir neurons soma size in relation to Iba1-ir microglial density and (**c**) relative masked area by AVP-ir cells in relation to Iba1-ir microglial density. Plots of (**d**) AVP-ir neurons density in relation to relative masked area of Iba1-ir microglial cells; (**e**) AVP-ir neurons soma size in relation to relative masked area of Iba1-ir microglial cells (**f**) AVP-ir relative masked area in relation to elative masked area of Iba1-ir microglial cells. AVP= arginine vasopressin; Iba1 = ionized calcium-binding adapter molecule 1; ir = immunoreactivity; PVN = paraventricular nucleus; CSF = cerebrospinal fluid; post-abs=post absorptive; T2DM = type 2 diabetic mellitus. Control: n = 20, T2DM: n = 26.

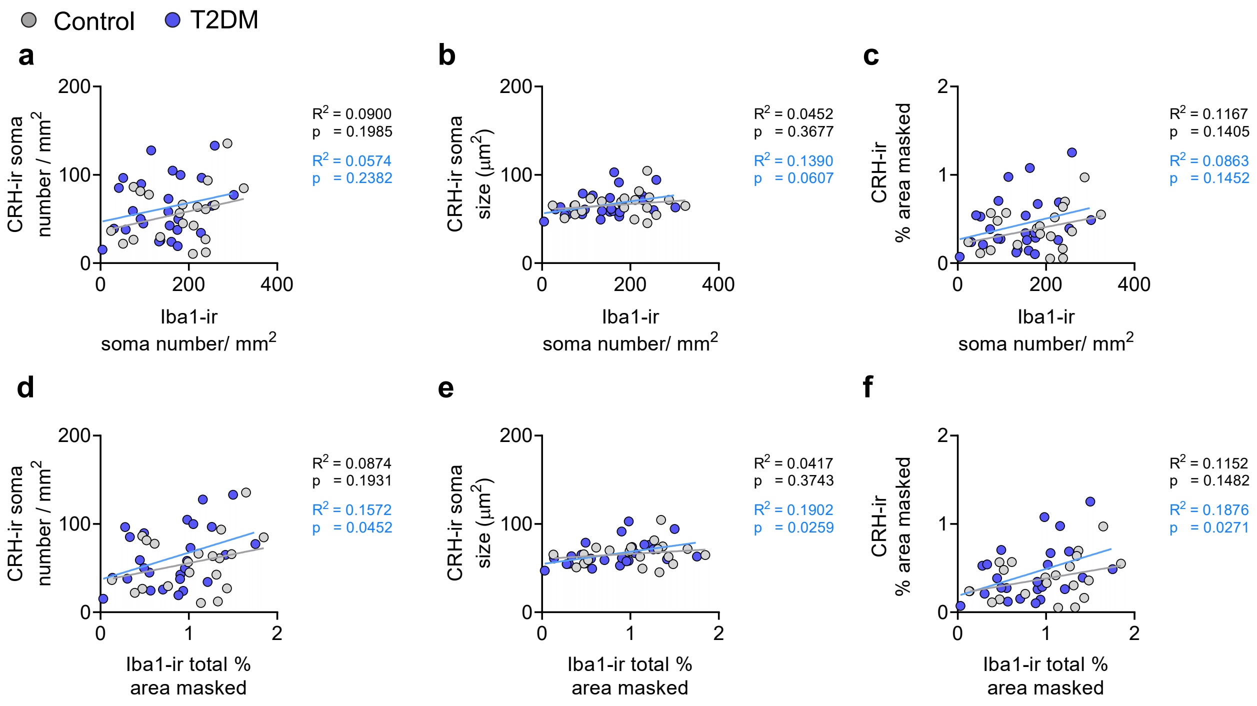

Supplementary Fig. 7

Analysis of corticotropin-ir (CRH-ir) neurons in relation to microglial (Iba1-ir) cells in the PVN of the hypothalamus of control and T2DM subjects. Plots of (**a**) CRH-ir neurons density in relation to Iba-ir microglial density; (**b**) CRH-ir neurons soma size in relation to Iba1-ir microglial density and (**c**) relative masked area by CRH-ir cells in relation to Iba1-ir microglial density. Plots of (**d**) CRH-ir neurons density in relation to relative masked area of Iba1-ir microglial cells; (**e**) CRH-ir neurons soma size in relation to relative masked area of Iba1-ir microglial cells (**f**) CRH-ir relative masked area in relation to elative masked area of Iba1-ir microglial cells. CRH = corticotropin releasing hormone; Iba1 = ionized calcium-binding adapter molecule 1; PVN = paraventricular nucleus; ir = immunoreactivity; CSF = cerebrospinal fluid; post-abs=post absorptive; T2DM = type 2 diabetic mellitus. Control: n = 20, T2DM: n = 26.

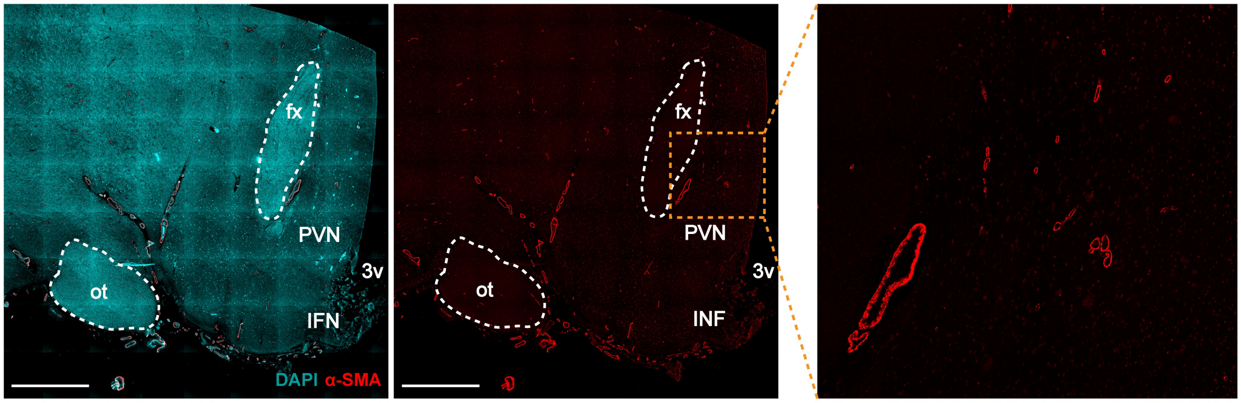

Supplementary Fig. 8

Representative image of alpha-smooth muscle actin-ir (alpha-SMA) in the human hypothalamus. Alpha-SMA and DAPI co-labelling (left image), single alpha-SMA channel (center). Yellow dashed-line partially frames the PVN, with provided higher magnification of alpha-SMA-ir pattern. 3V = third ventricle; fx =fornix; IFN = infundibular nucleus; PVN = paraventricular nucleus; ot = optic tract. Scale bar = 2 mm.

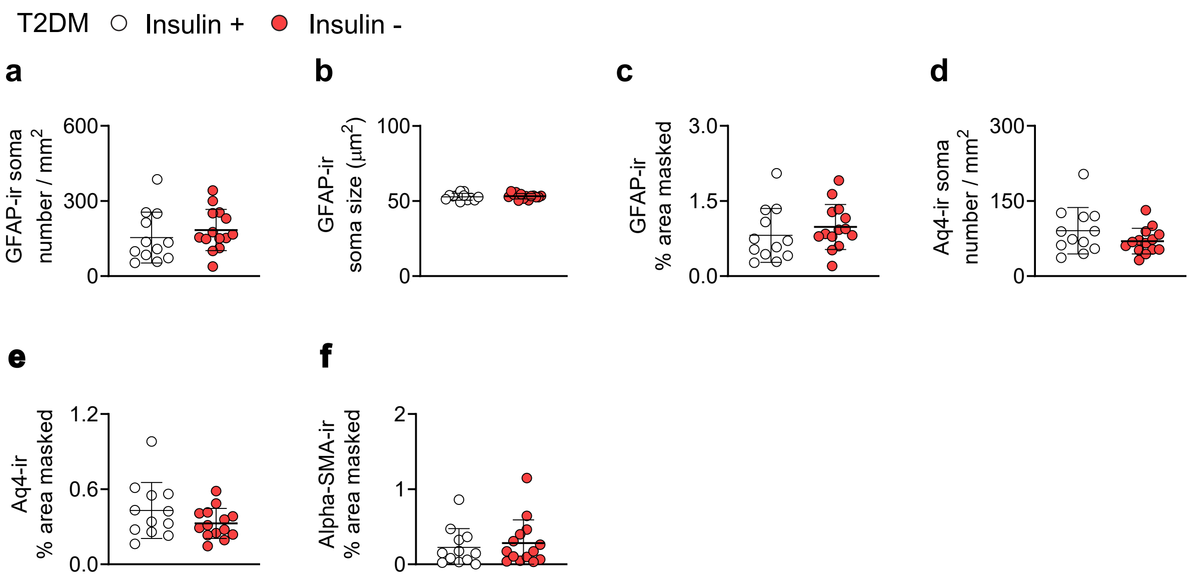

Supplementary Fig. 9

The antidiabetic treatment-associated effect on PVN astroglia and glymphatic system. Comparison between T2DM individuals with or without insulin treatment. (**a**) GFAP-ir soma number / mm^2 (cell density), (**b**) GFAP-ir average soma size and (**c**) relative masked area by positive astrocytic particles. (**d**) Quantitative analysis of Aq4-ir cells / mm^2 (density) and (**e**) relative masked area by Aq4-ir. (**f**) Quantitative analysis of alpha-SMA-ir relative masked area by positive endothelial cells. Please note that astrocytic and glymphatic system components are not affected by insulin treatment in T2DM subjects. GFAP = glial fibrillary acid protein; Aq4 = aquaporin 4; alpha-SMA = alpha smooth muscle actin; ir = immunoreactivity; PVN = paraventricular nucleus; T2DM = type 2 diabetic mellitus. Data are represented as mean ± SEM. T2DM insulin + : n = 12, T2DM insulin – : n=14.

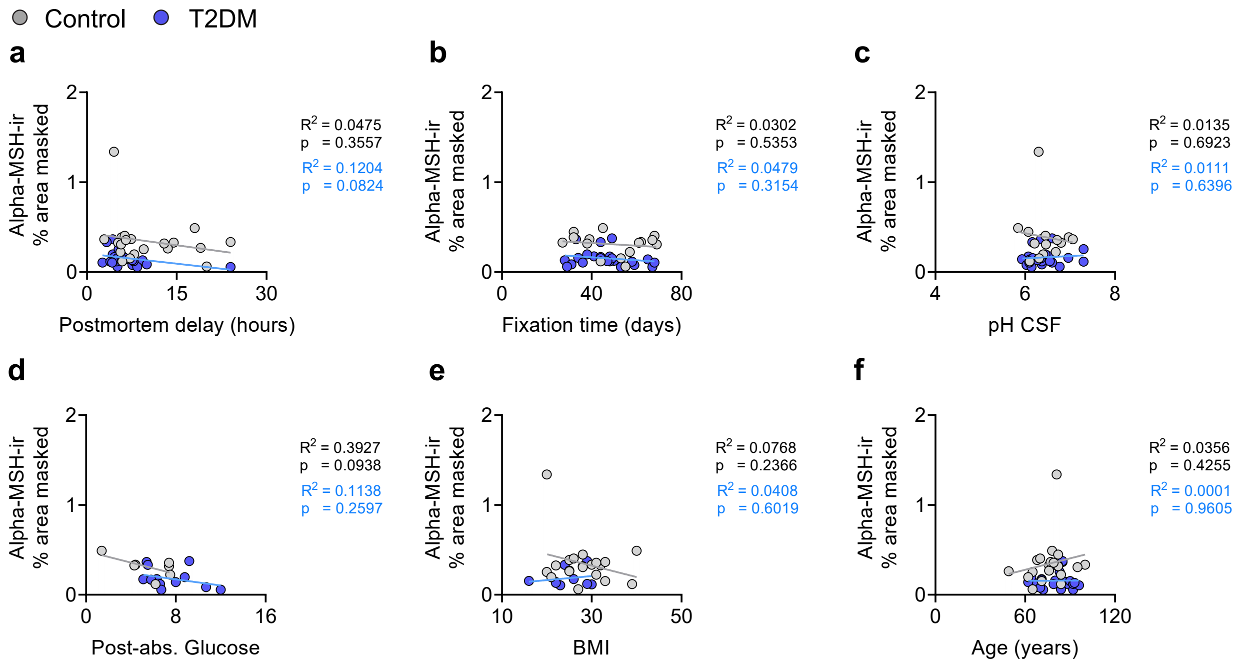

Supplementary Fig. 10

Co-founder analysis of alpha-MSH-ir fibers area masked in the PVN of control and T2DM subjects. (**a-f**) Plots of alpha-MSH-ir relative masked area in the PVN according to (**a**) postmortem delay, (**b**) fixation time, (**c**) pH of CSF, (**d**) post absorptive glucose, (**e**) and body mass index (BMI) and (**f**) age. Alpha-MSH = alpha-melanocortin stimulating hormone; PVN = paraventricular nucleus; ir = immunoreactivity; CSF = cerebrospinal fluid; post-abs=post absorptive; T2DM = type 2 diabetic mellitus. Control: n = 20, T2DM: n = 26.

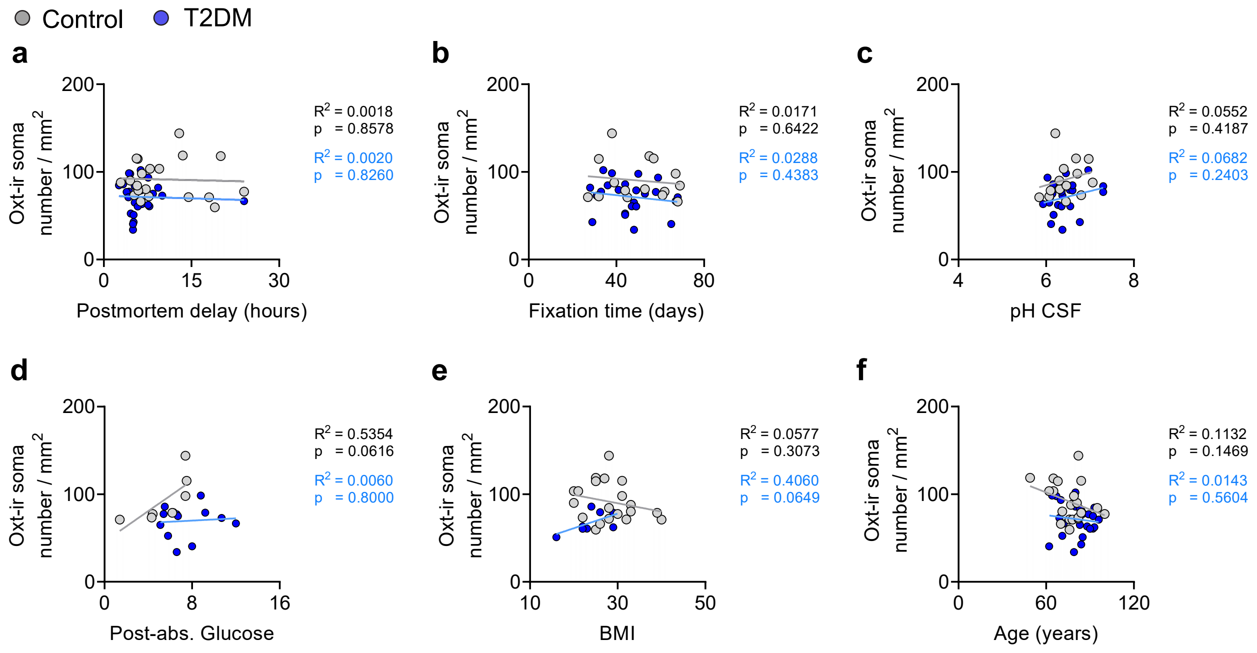

Supplementary Fig. 11

Co-founder analysis of oxytocin-ir (Oxt-ir) neurons density in the PVN of control and T2DM subjects. (**a-f**) Plots of Oxt-ir soma number/mm2 in the PVN according to (**a**) postmortem delay, (**b**) fixation time, (c) pH of CSF, (**d**) post absorptive glucose, (**e**) and body mass index (BMI) and (**f**) age. Oxt = oxytocin; PVN = paraventricular nucleus; ir = immunoreactivity; CSF = cerebrospinal fluid; post-abs=post absorptive; T2DM = type 2 diabetic mellitus. Control: n = 20, T2DM: n = 26.

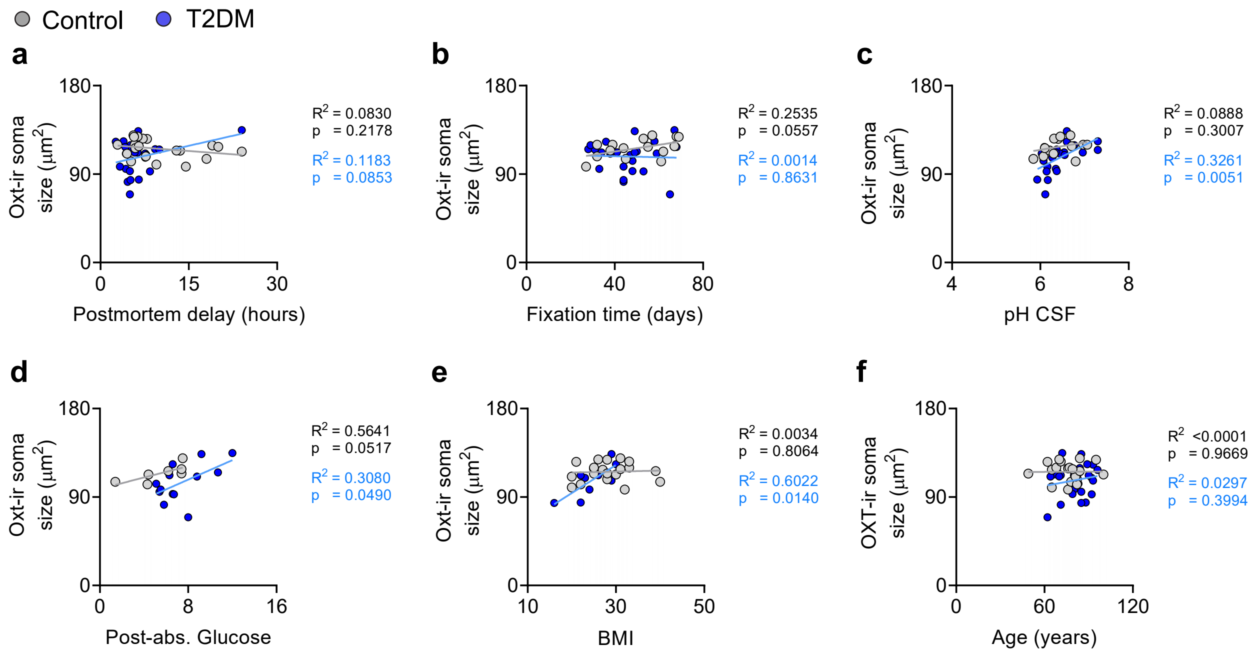

Supplementary Fig. 12

Co-founder analysis of oxytocin-ir (Oxt-ir) soma size in the PVN of control and T2DM subjects. (**a-f**) Plots of oxytocin-ir soma size in the PVN according to (**a**) postmortem delay, (**b**) fixation time, (**c**) pH of CSF, (**d**) post absorptive glucose, (**e**) and body mass index (BMI) and (**f**) age. Oxt = oxytocin; PVN = paraventricular nucleus; ir = immunoreactivity; CSF = cerebrospinal fluid; post-abs=post absorptive; T2DM = type 2 diabetic mellitus. Control: n = 20, T2DM: n = 26.

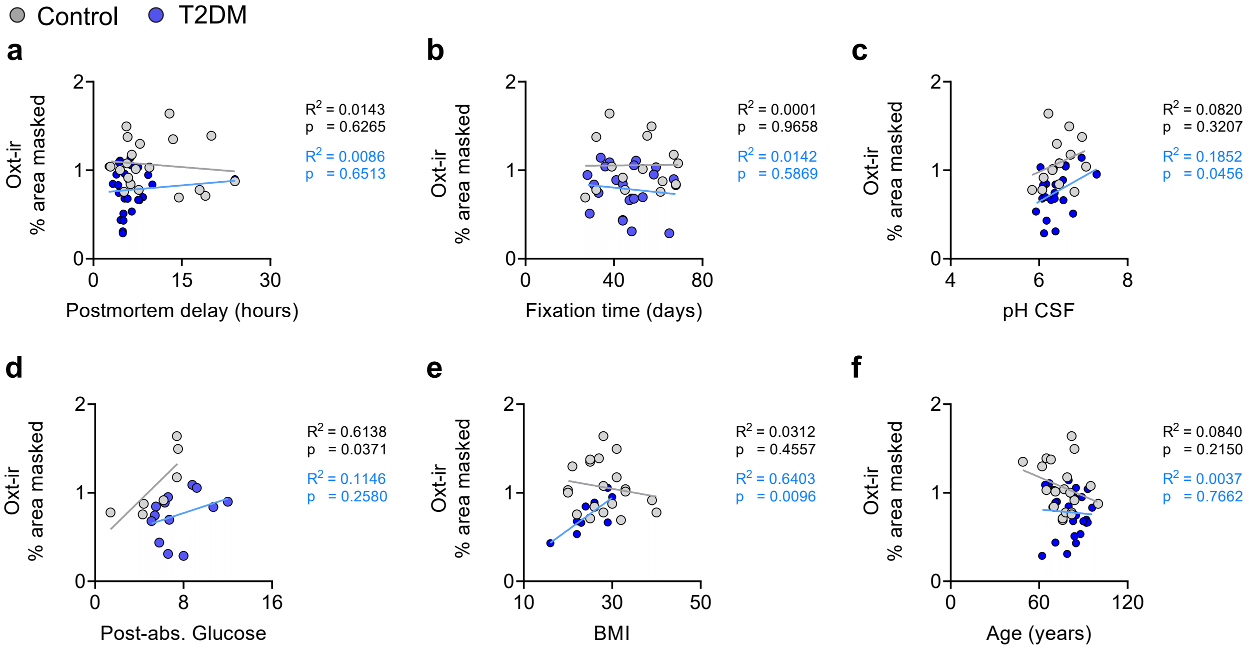

Supplementary Fig. 13

Co-founder analysis of total area masked by positive oxytocin-ir (Oxt-ir) cells in the PVN of control and T2DM subjects. (**a**-**f**) Plots of xt-ir relative masked area in the PVN according to (**a**) postmortem delay, (**b**) fixation time, (**c**) pH of CSF, (**d**) post absorptive glucose, (**e**) and body mass index (BMI) and (**f**) age. Oxt = oxytocin; ir = immunoreactivity; CSF = cerebrospinal fluid; post-abs=post absorptive; T2DM = type 2 diabetic mellitus. Control: n = 20, T2DM: n = 26.

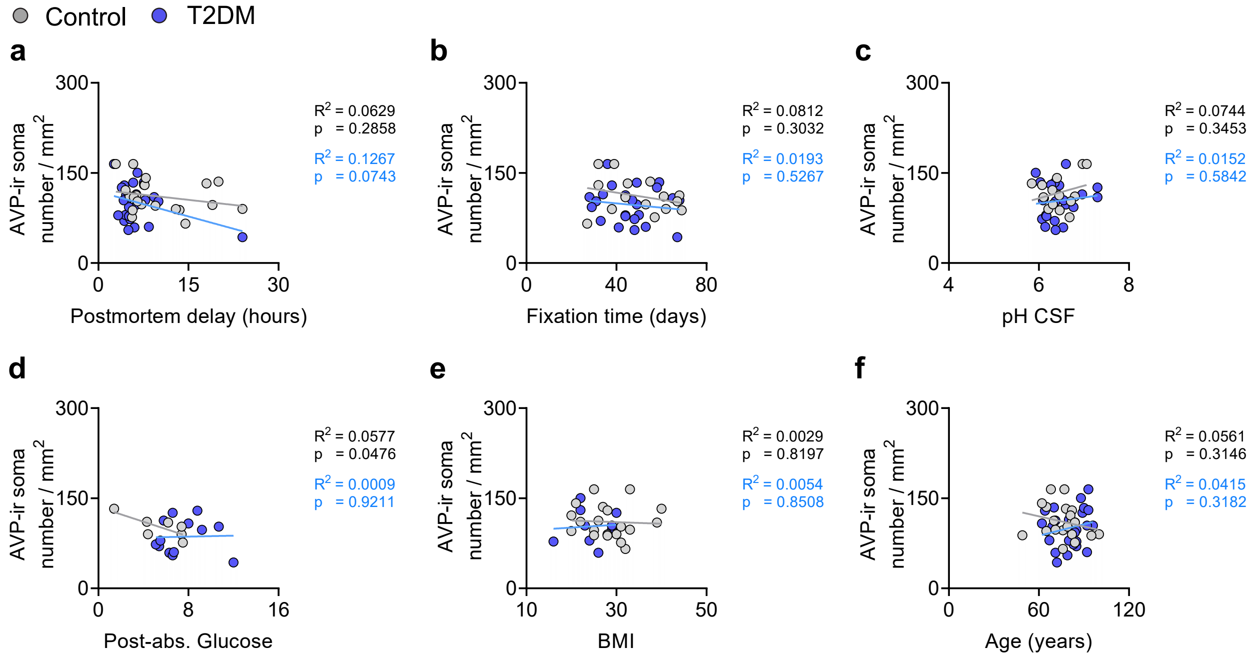

Supplementary Fig. 14

Co-founder analysis of arginine-vasopressin-ir (AVP-ir) neurons density in the PVN of control and T2DM subjects. (**a**-**f**) Plots of AVP-ir soma number/mm2 in the PVN according to (**a**) postmortem delay, (**b**) fixation time, (**c**) pH of CSF, (**d**) post absorptive glucose, (**e**) and body mass index (BMI) and (**f**) age. AVP = arginine-vasopressin; PVN = paraventricular nucleus; ir = immunoreactivity; CSF = cerebrospinal fluid; post-abs=post absorptive; T2DM = type 2 diabetic mellitus. Control: n = 20, T2DM: n = 26.

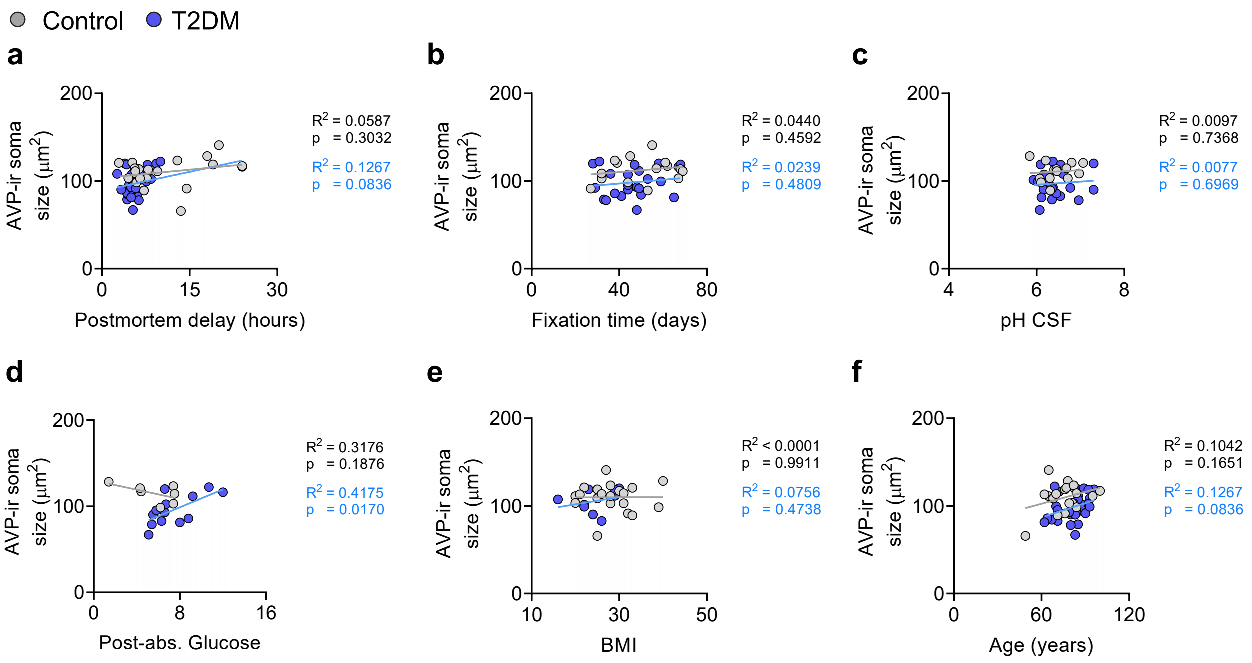

Supplementary Fig. 15

Co-founder analysis of arginine-vasopressin-ir (AVP-ir) soma size in the PVN of control and T2DM subjects. (**a**-**f**) Plots of AVP-ir soma size in the PVN according to (**a**) postmortem delay, (b) fixation time, (**c**) pH of CSF, (**d**) post absorptive glucose, (**e**) and body mass index (BMI) and (**f**) age. AVP = arginine-vasopressin; PVN = paraventricular nucleus; ir = immunoreactivity; CSF = cerebrospinal fluid; post-abs=post absorptive; T2DM = type 2 diabetic mellitus. Control: n = 20, T2DM: n = 26.

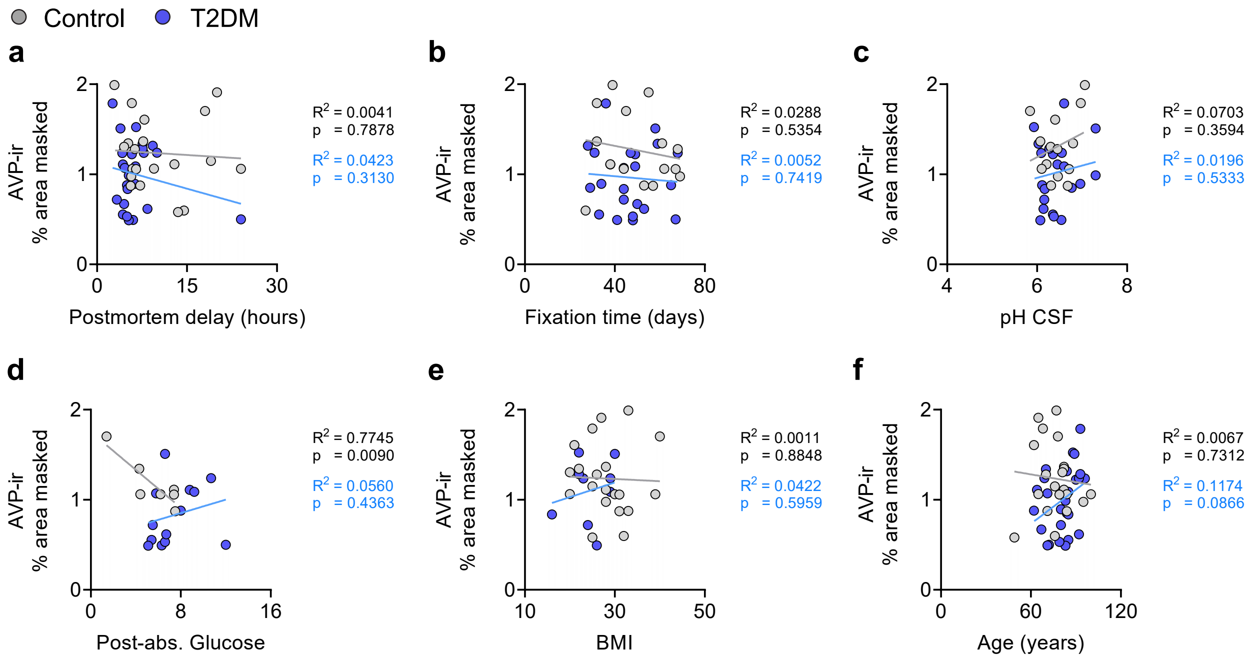

Supplementary Fig. 16

Co-founder analysis of total area masked by positive arginine-vasopressin-ir (AVP-ir) cells in the PVN of control and T2DM subjects. (**a**-**f**) Plots of AVP-ir relative masked area in the PVN according to (**a**) postmortem delay, (**b**) fixation time, (**c**) pH of CSF, (**d**) post absorptive glucose, (**e**) and body mass index (BMI) and (**f**) age. AVP = arginine-vasopressin; PVN = paraventricular nucleus; ir = immunoreactivity; CSF = cerebrospinal fluid; post-abs=post absorptive; T2DM = type 2 diabetic mellitus. Control: n = 20, T2DM: n = 26.

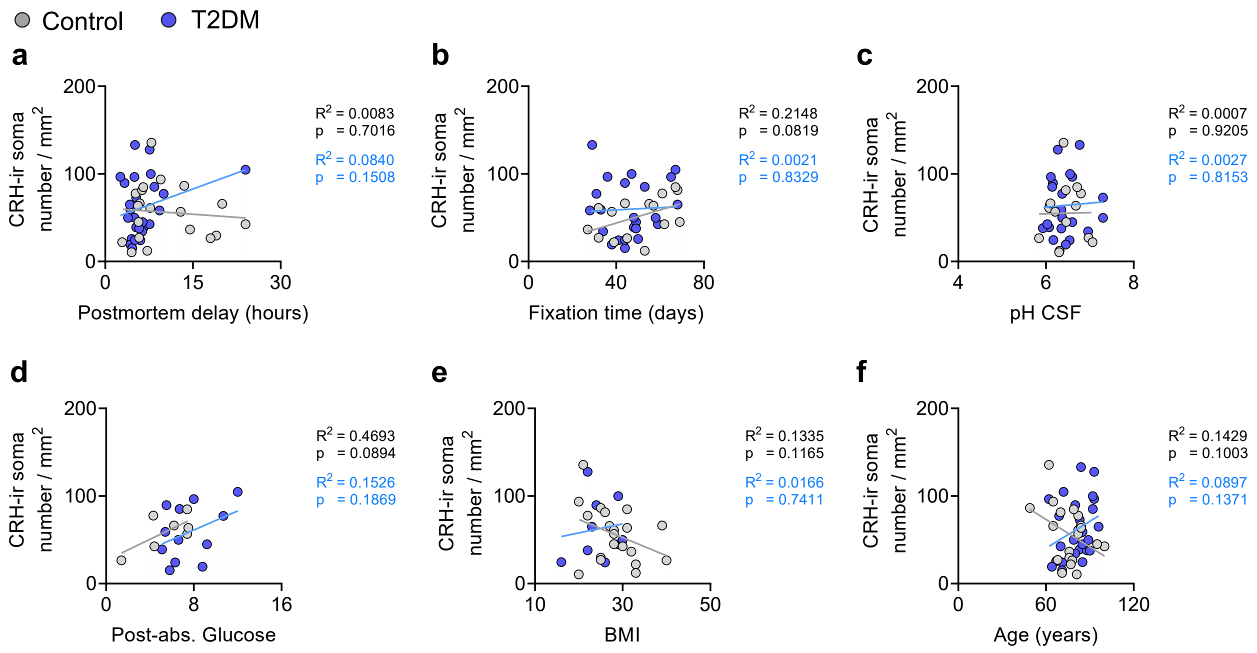

Supplementary Fig. 17

Co-founder analysis of corticotropin releasing hormone-ir (CRH-ir) neurons density in the PVN of control and T2DM subjects. (**a**-**f**) Plots of CRH-ir soma number/mm2 in the hypothalamic PVN according to (**a**) postmortem delay, (**b**) fixation time, (**c**) pH of CSF, (**d**) post absorptive glucose, (**e**) and body mass index (BMI) and (**f**) age. CRH = corticotropin releasing hormone; PVN = paraventricular nucleus; ir = immunoreactivity; CSF = cerebrospinal fluid; post-abs=post absorptive; T2DM = type 2 diabetic mellitus. Control: n = 20, T2DM: n = 26.

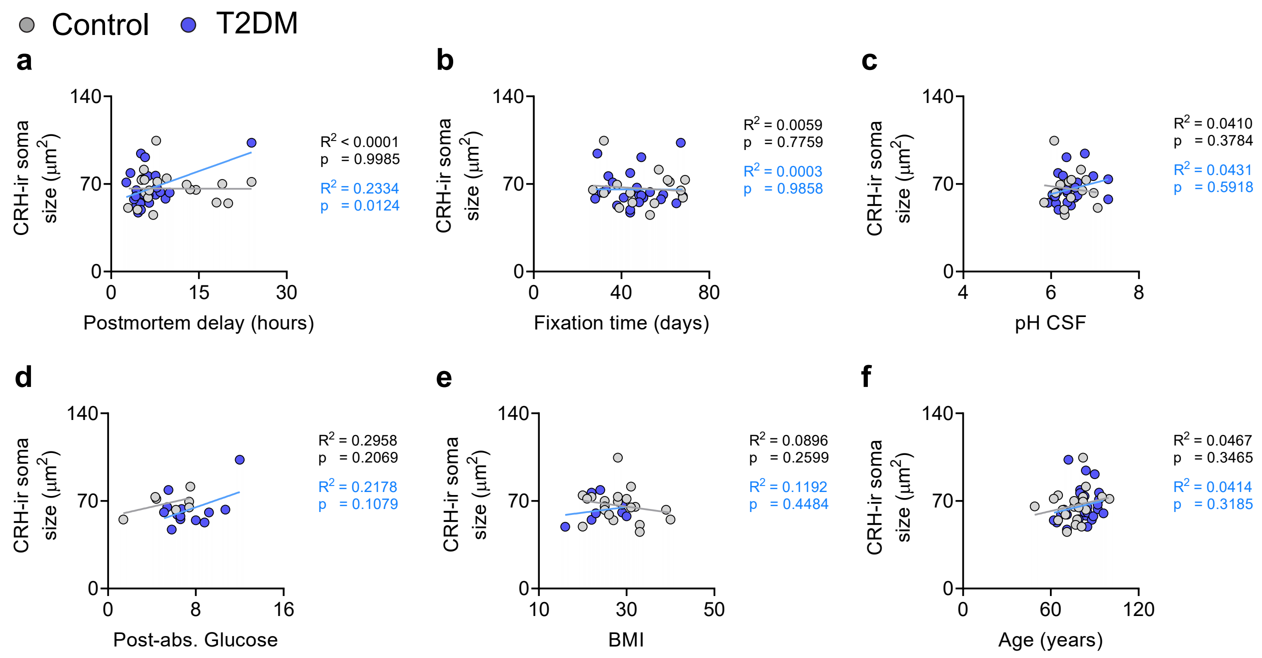

Supplementary Fig. 18

Co-founder analysis of corticotropin releasing hormone-ir (CRH-ir) soma size in the PVN of control and T2DM subjects. (**a**-**f**) Plots of CRH-ir soma size in the PVN according to (**a**) postmortem delay, (**b**) fixation time, (**c**) pH of CSF, (**d**) post absorptive glucose, (**e**) and body mass index (BMI) and (**f**) age. CRH = corticotropin releasing hormone; PVN = paraventricular nucleus; ir = immunoreactivity; CSF = cerebrospinal fluid; post-abs=post absorptive; T2DM = type 2 diabetic mellitus. Control: n = 20, T2DM: n = 26.

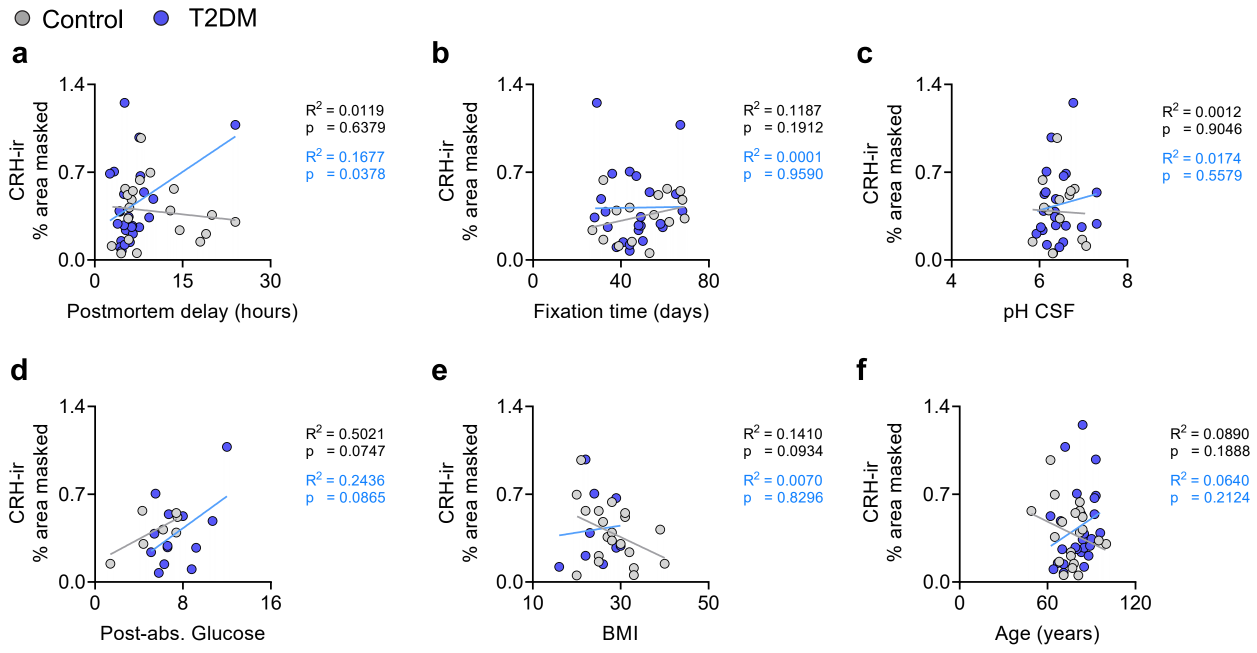

Supplementary Fig. 19

Co-founder analysis of total area masked by positive corticotropin releasing hormone-ir (CRH-ir) cells in the PVN of control and T2DM subjects. (**a**-**f**) Plots of CRH-ir relative masked area in the PVN according to (**a**) postmortem delay, (**b**) fixation time, (**c**) pH of CSF, (**d**) post absorptive glucose, (**e**) and body mass index (BMI) and (**f**) age. CRH = corticotropin releasing hormone; PVN = paraventricular nucleus; ir = immunoreactivity; CSF = cerebrospinal fluid; post-abs=post absorptive; T2DM = type 2 diabetic mellitus. Control: n = 20, T2DM: n = 26.

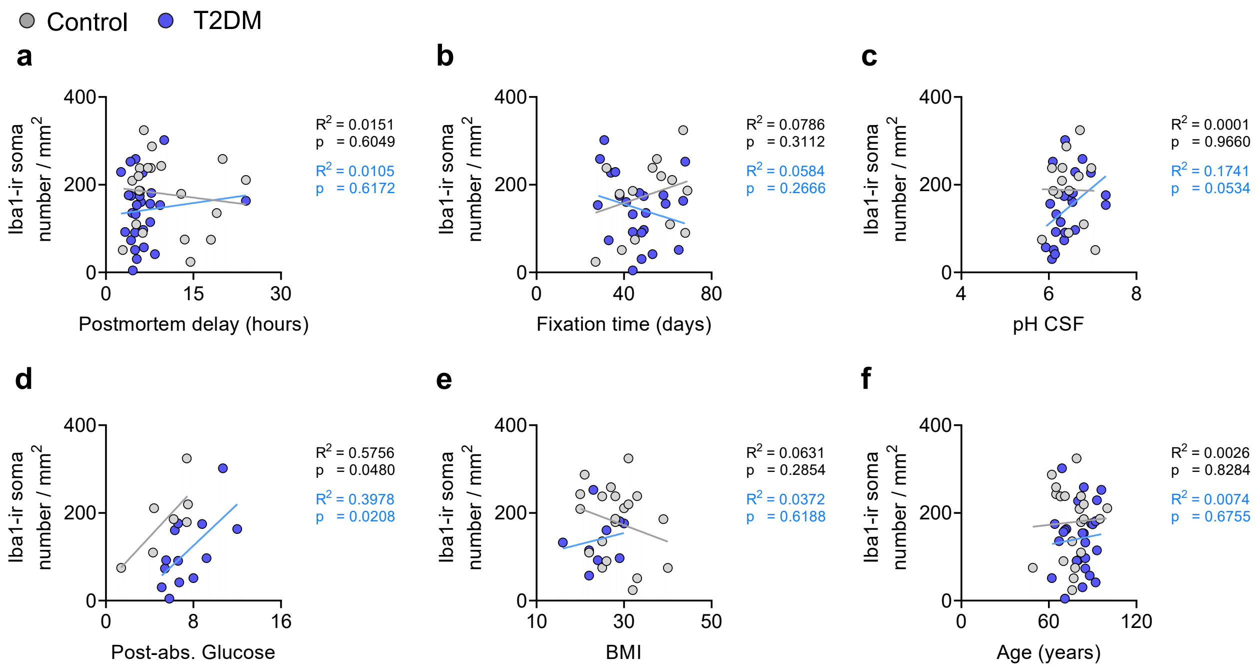

Supplementary Fig. 20

Co-founder analysis of microglial (Iba1-ir) density in the PVN of control and T2DM subjects. (**a-f**) Plots of Iba1-ir soma number/mm2 in the PVN according to (**a**) postmortem delay, (**b**) fixation time, (**c**) pH of CSF, (**d**) post absorptive glucose, (**e**) and body mass index (BMI) and (**f**) age. Iba1 = ionized calcium-binding adapter molecule 1; ir = immunoreactivity; CSF = cerebrospinal fluid; post-abs=post absorptive; T2DM = type 2 diabetic mellitus. Control: n = 20, T2DM: n = 26.

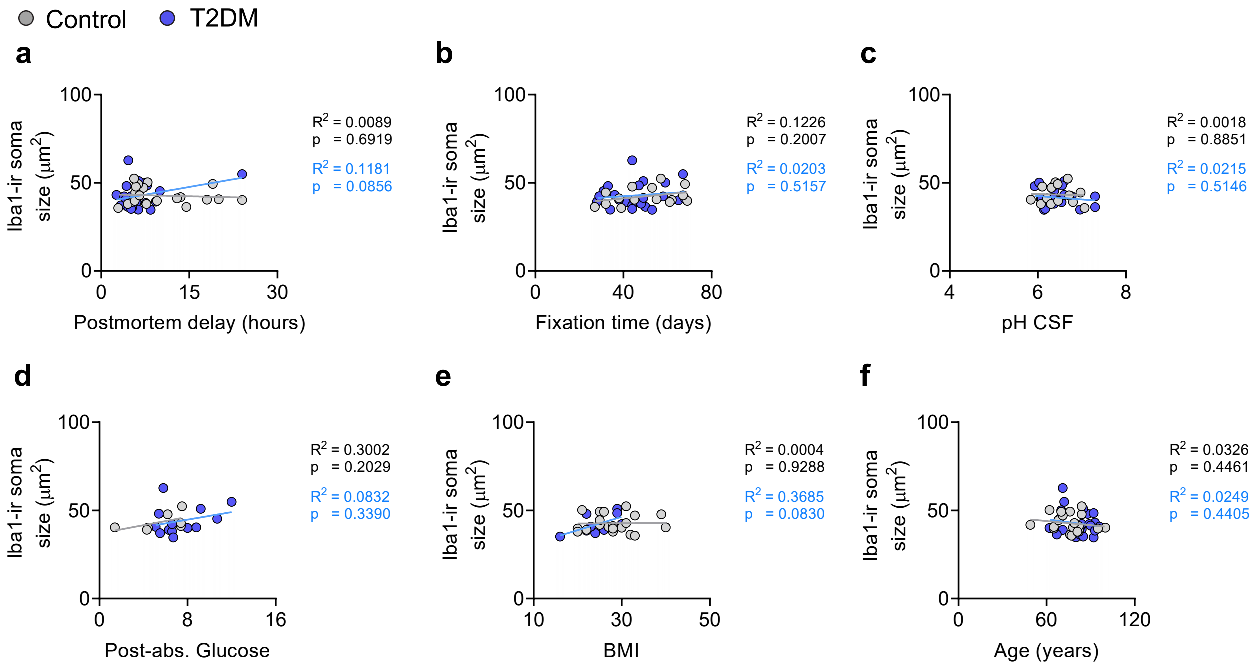

Supplementary Fig. 21

Co-founder analysis of microglial (Iba1-ir) soma size in the PVN of control and T2DM subjects. (**a-f**) Plots of Iba1-ir soma size in the PVN according to (**a**) postmortem delay, (**b**) fixation time, (**c**) pH of CSF, (**d**) post absorptive glucose, (**e**) and body mass index (BMI) and (**f**) age. Iba1 = ionized calcium-binding adapter molecule 1; PVN = paraventricular nucleus; ir = immunoreactivity; CSF = cerebrospinal fluid; post-abs=post absorptive; T2DM = type 2 diabetic mellitus. Control: n = 20, T2DM: n = 26.

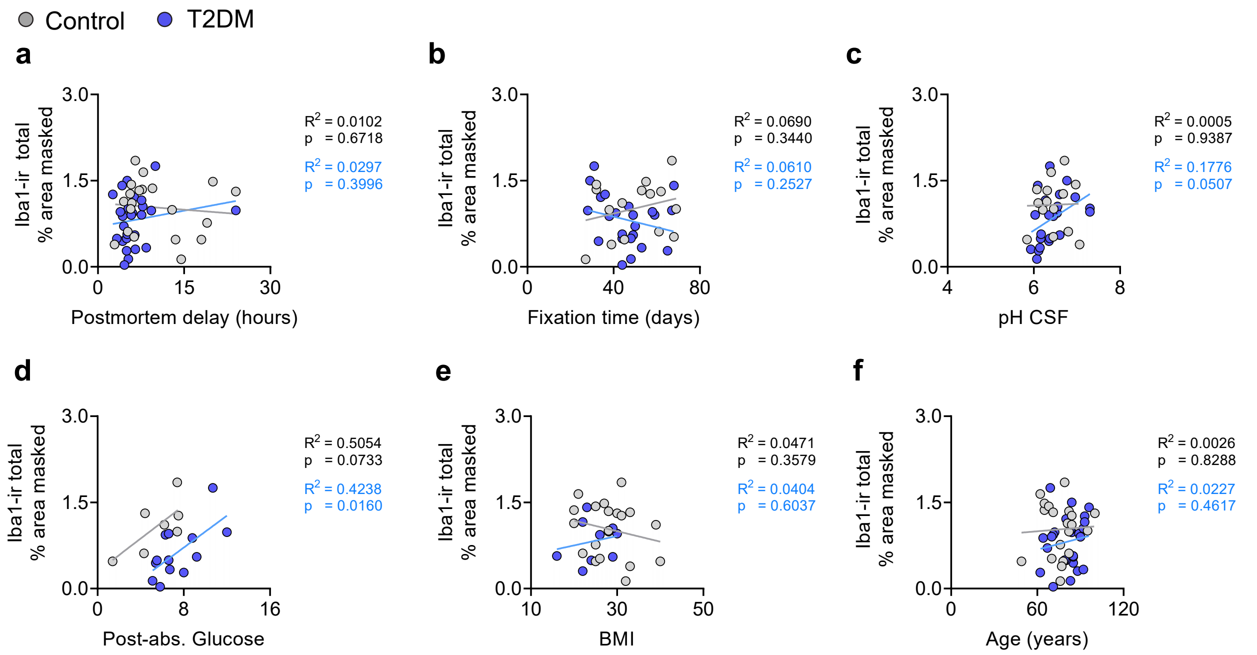

Supplementary Fig. 22

Co-founder analysis of total area masked by Iba1-ir microglial cells in the PVN of control and T2DM subjects. (**a**-**f**) Plots of Iba1-ir relative masked area in the PVN according to (**a**) postmortem delay, (**b**) fixation time, (**c**) pH of CSF, (**d**) post absorptive glucose, (**e**) and body mass index (BMI) and (**f**) age. Iba1 = ionized calcium-binding adapter molecule 1; PVN = paraventricular nucleus; ir = immunoreactivity; CSF = cerebrospinal fluid; post-abs=post absorptive; T2DM = type 2 diabetic mellitus. Control: n = 20, T2DM: n = 26.

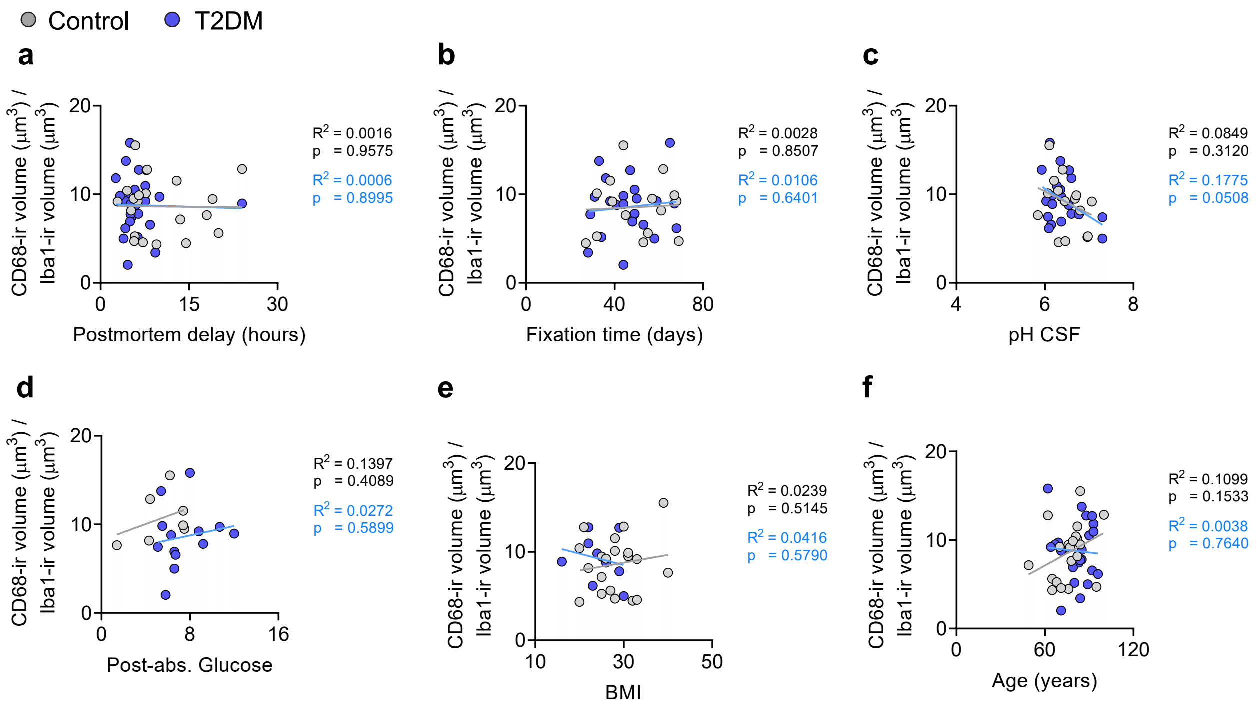

Supplementary Fig. 23

Co-founder analysis of microglial cluster of differentiation-ir (CD68-ir) in the PVN of control and T2DM subjects. (**a**-**f**) Plots of volume percentage of CD68-ir in relation to Iba1-ir in the PVN according to (**a**) postmortem delay, (**b**) fixation time, (**c**) pH of CSF, (**d**) post absorptive glucose, (**e**) and body mass index (BMI) and (**f**) age. CD68 = cluster of differentiation 68; Iba1 = ionized calcium-binding adapter molecule 1; PVN = paraventricular nucleus; ir = immunoreactivity; CSF = cerebrospinal fluid; post-abs=post absorptive; T2DM = type 2 diabetic mellitus. Control: n = 20, T2DM: n = 26.

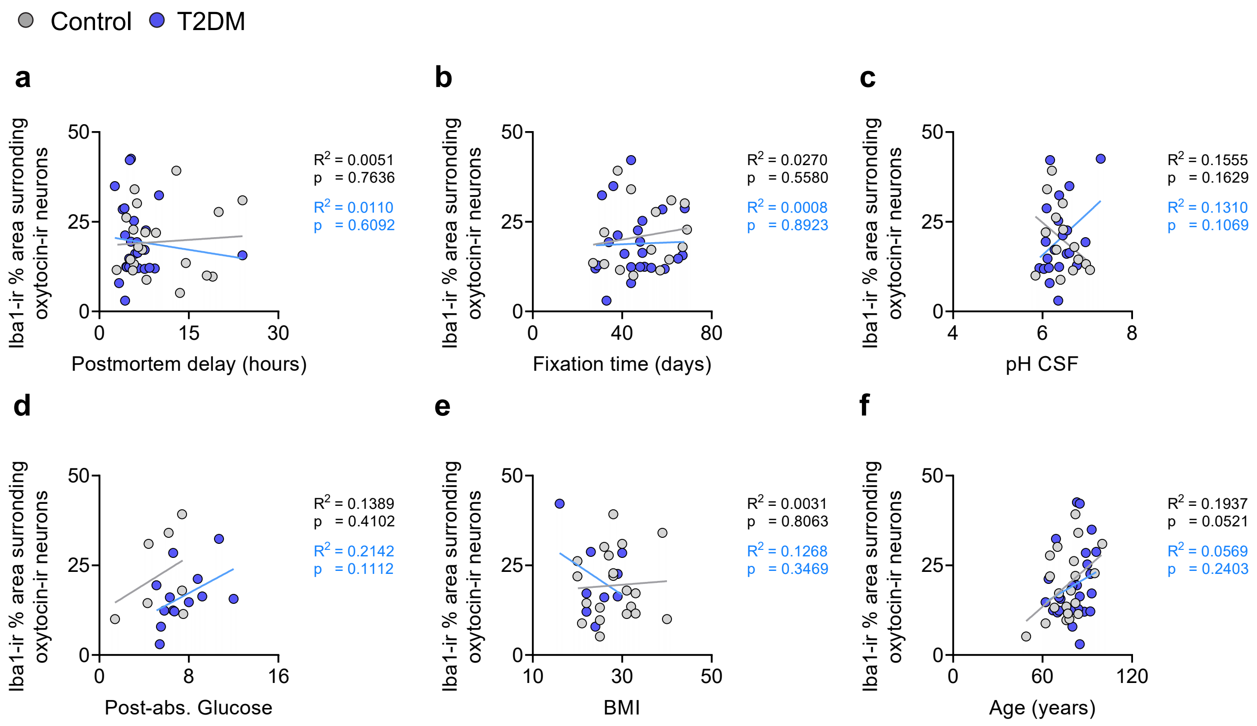

Supplementary Fig. 24

Co-founder analysis of mean relative area masked (%) of Iba1-ir particles surrounding oxytocin-ir (Oxt-ir) neurons in the PVN of control and T2DM subjects. (**a-f**) Plots of Iba1-ir relative masked area surrounding Oxt-ir neurons (within 10 µm radius) in the PVN according to (**a**) postmortem delay, (**b**) fixation time, (**c**) pH of CSF, (**d**) post absorptive glucose, (**e**) and body mass index (BMI) and (**f**) age. (Iba1 = ionized calcium-binding adapter molecule 1; Oxt = oxytocin; ir = immunoreactivity; CSF = cerebrospinal fluid; post-abs=post absorptive; T2DM = type 2 diabetic mellitus. Control: n = 20, T2DM: n = 26.

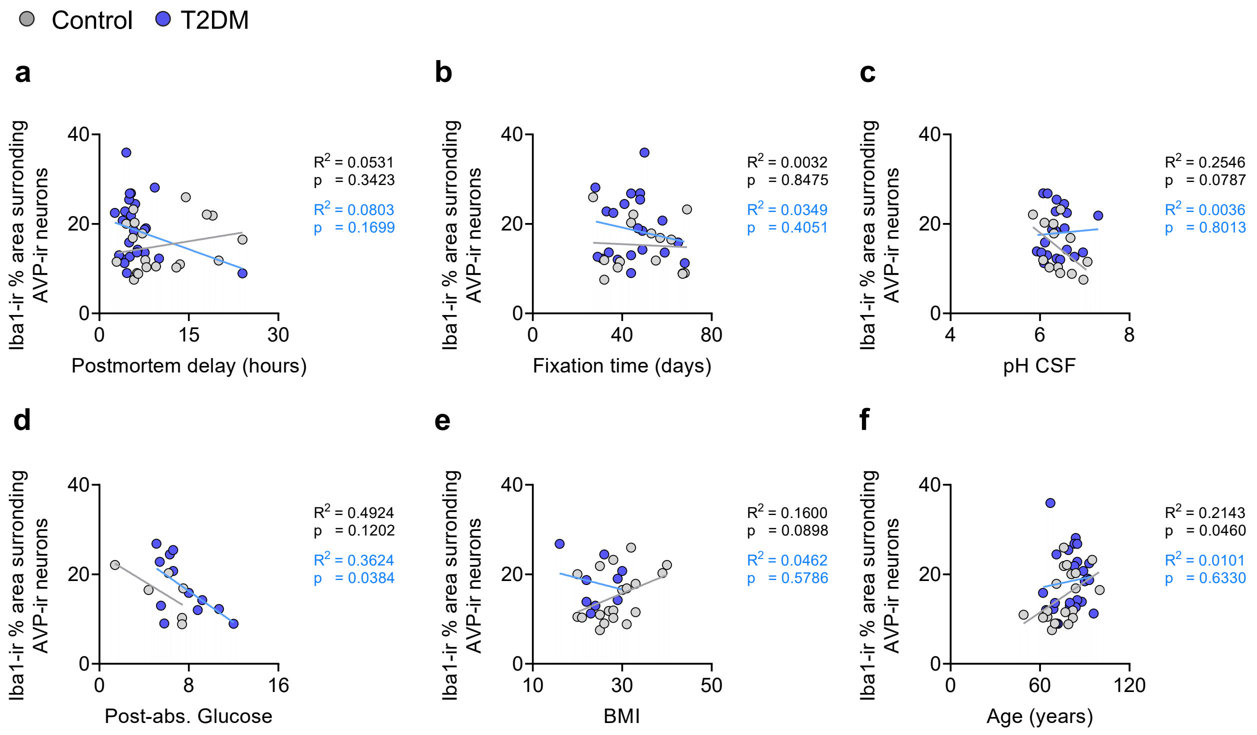

Supplementary Fig. 25

Co-founder analysis of mean relative area masked (%) of Iba1-ir particles surrounding AVP-ir neurons in the PVN of control and T2DM subjects. (**a-f**) Plots of Iba1-ir relative masked area surrounding AVP-ir neurons (within 10 µm radius) in the PVN according to (**a**) postmortem delay, (**b**) fixation time, (**c**) pH of CSF, (**d**) post absorptive glucose, (**e**) and body mass index (BMI) and (**f**) age. Iba1 = ionized calcium-binding adapter molecule 1; AVP = arginine-vasopressin; PVN = paraventricular nucleus; ir = immunoreactivity; CSF = cerebrospinal fluid; post-abs=post absorptive; T2DM = type 2 diabetic mellitus. Control: n = 20, T2DM: n = 26.

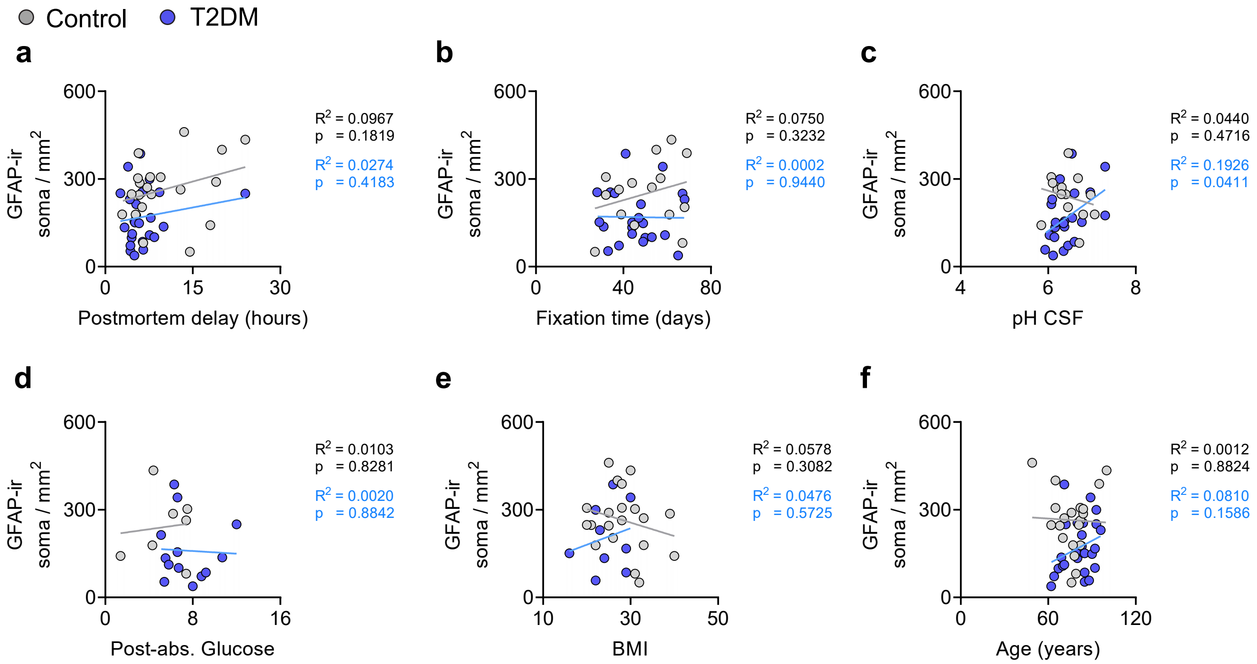

Supplementary Fig. 26

Co-founder analysis of astroglial (GFAP-ir) density in the PVN of control and T2DM subjects. (**a-f**) Plots of GFAP-ir soma number/mm2 in the PVN according to (**a**) postmortem delay, (**b**) fixation time, (**c**) pH of CSF, (**d**) post absorptive glucose, (**e**) and body mass index (BMI) and (**f**) age. GFAP = glial fibrillary acid protein; ir = immunoreactivity; PVN = paraventricular nucleus; CSF = cerebrospinal fluid; post-abs=post absorptive; T2DM = type 2 diabetic mellitus. Control: n = 20, T2DM: n = 26.

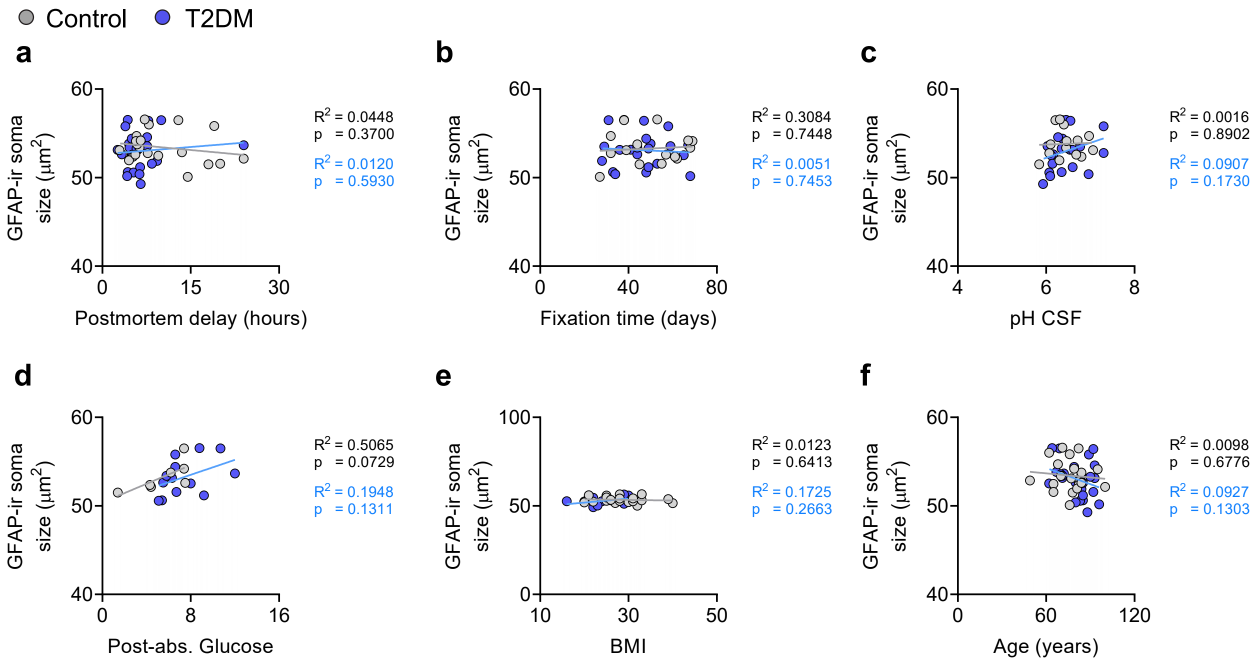

Supplementary Fig. 27

Co-founder analysis of astroglial (GFAP-ir) soma size in the PVN of control and T2DM subjects. (**a-f**) Plots of GFAP-ir soma size in the PVN according to (**a**) postmortem delay, (**b**) fixation time, (**c**) pH of CSF, (**d**) post absorptive glucose, (**e**) and body mass index (BMI) and (**f**) age. GFAP = glial fibrillary acid protein; ir = immunoreactivity; PVN = paraventricular nucleus; CSF = cerebrospinal fluid; post-abs=post absorptive; T2DM = type 2 diabetic mellitus. Control: n = 20, T2DM: n = 26.

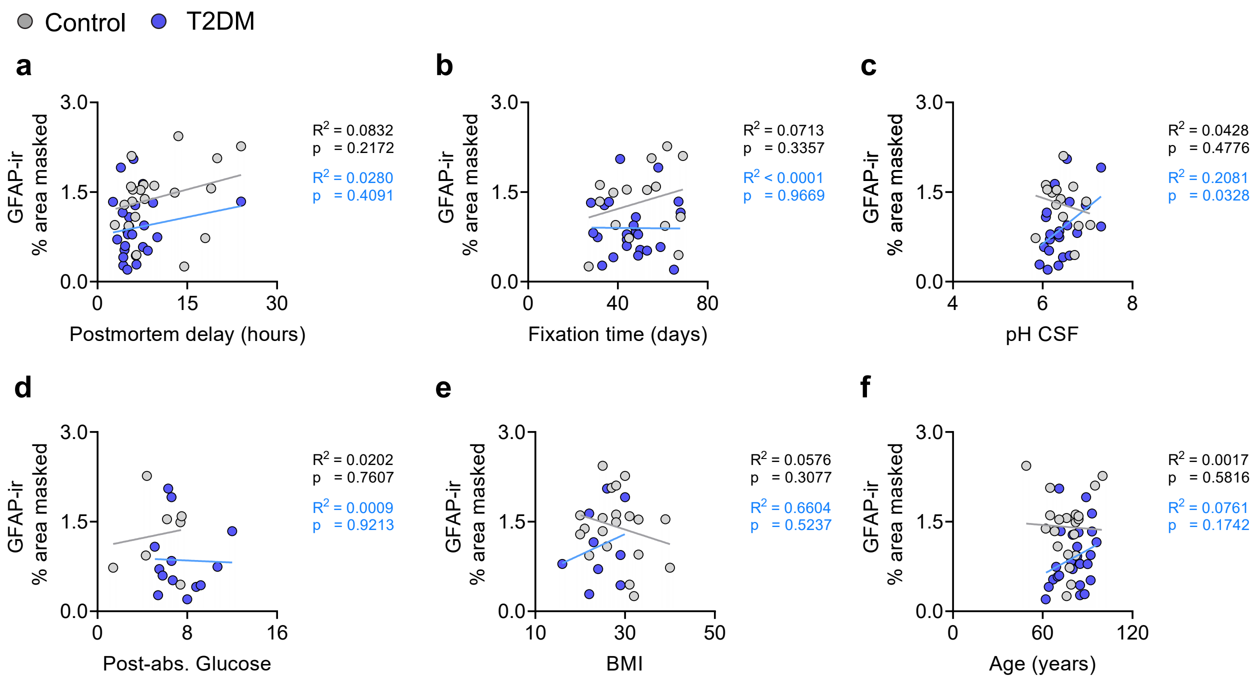

Supplementary Fig. 28

Co-founder analysis of astroglial (GFAP-ir) relative masked area in the PVN of control and T2DM subjects. (**a**-**f**) Plots of GFAP-ir relative masked area in the PVN according to (**a**) postmortem delay, (**b**) fixation time, (**c**) pH of CSF, (**d**) post absorptive glucose, (**e**) and body mass index (BMI) and (**f**) age. GFAP = glial acid fibrillary acid protein; PVN = paraventricular nucleus; ir = immunoreactivity; CSF = cerebrospinal fluid; post-abs=post absorptive; T2DM = type 2 diabetic mellitus. Control: n = 20, T2DM: n = 26.

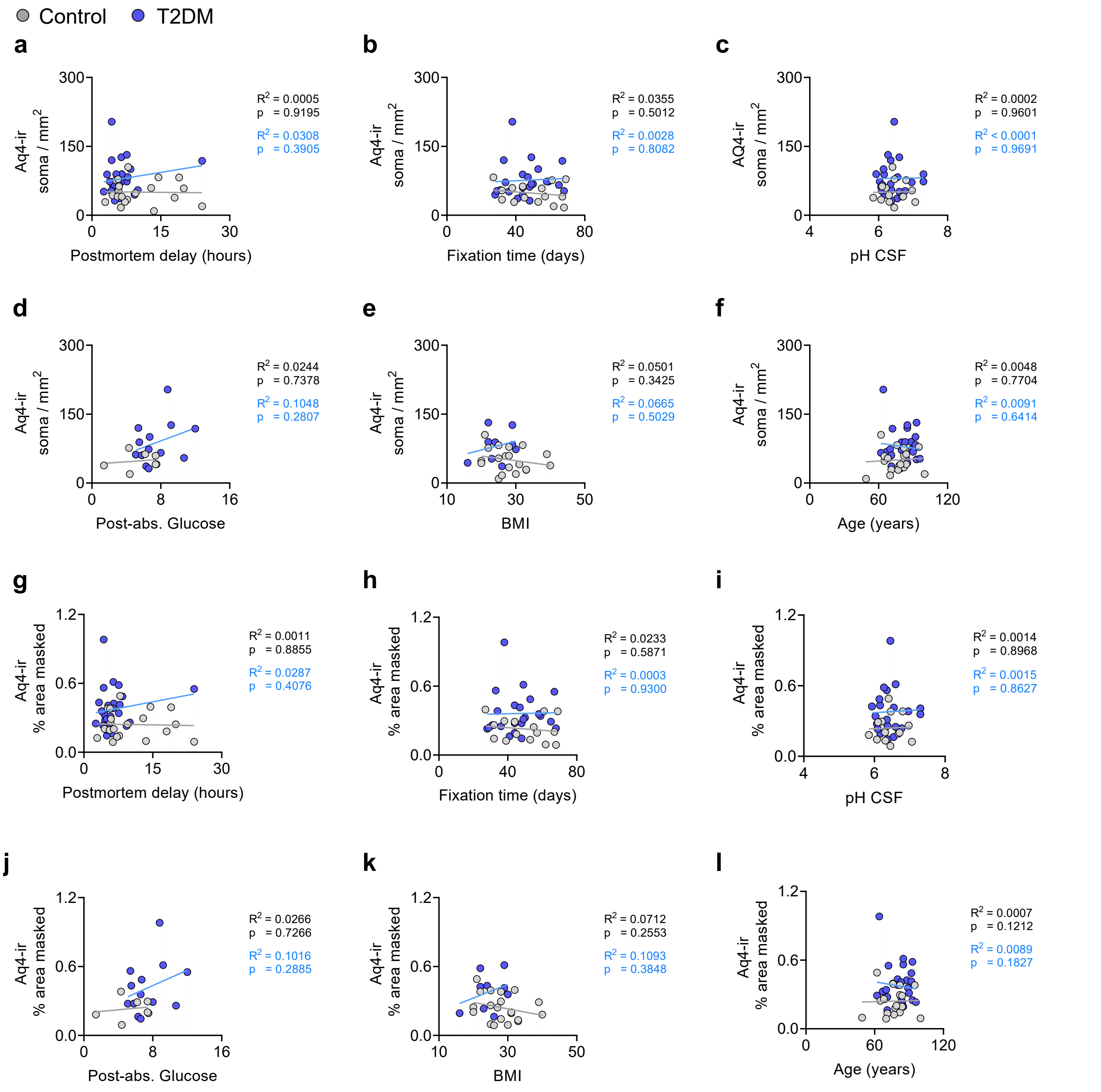

Supplementary Fig. 29

Co-founder analysis of aquaporin 4-ir astrocytes (Aq4-ir) in the PVN of control and T2DM subjects. (**a-f**) Plots of Aq4-ir soma number/mm2 in the PVN according to (**a**) postmortem delay, (**b**) fixation time, (**c**) pH of CSF, (**d**) post absorptive glucose, (**e**) and body mass index (BMI) and (**f**) age. (**g-l**) Plots of AQ4-ir relative masked area in PVN according to (**g**) postmortem delay, (**h**) fixation time, (**i**) pH of CSF, (**j**) post absorptive glucose, (**k**) and body mass index (BMI) and (**l**) age. Aq4 = aquaporin 4; PVN = paraventricular nucleus; ir = immunoreactivity; CSF = cerebrospinal fluid; post-abs=post absorptive; T2DM = type 2 diabetic mellitus. Control: n = 20, T2DM: n = 26.

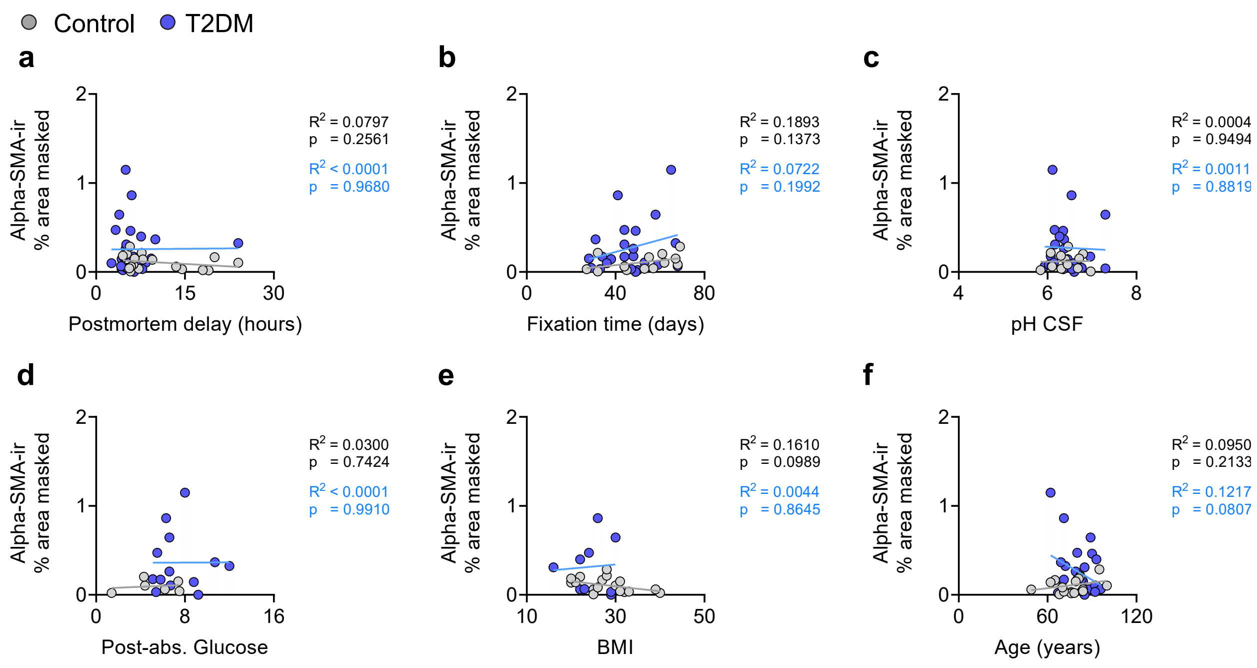

Supplementary Fig. 30

Co-founder analysis of vasculature (alpha-SMA-ir) relative masked area in the PVN of control and T2DM subjects. (**a**-**f**) Plots of alpha-SMA-ir relative masked areain the PVN according to (**a**) postmortem delay, (**b**) fixation time, (**c**) pH of CSF, (**d**) post absorptive glucose, (**e**) and body mass index (BMI) and (**f**) age. αSMA = alpha smooth muscle actin; PVN = paraventricular nucleus; ir = immunoreactivity; CSF = cerebrospinal fluid; post-abs=post absorptive; T2DM = type 2 diabetic mellitus. Control: n = 20, T2DM: n = 26.
